# Developing a toolkit for implementing evidence-based guidelines to manage hypertension and diabetes in Cambodia: a descriptive case study

**DOI:** 10.1186/s12961-022-00912-4

**Published:** 2022-11-29

**Authors:** Nirmali Sivapragasam, David B. Matchar, Pheak Chhoun, Hero Kol, Chhun Loun, Amina Mahmood Islam, John Ansah, Siyan Yi

**Affiliations:** 1 Programme in Health Services and Systems Research, Duke-NUS Medical School, 8 College Road, Singapore, 169857 Singapore; 2 Duke University Medical Center, Duke University, Durham, NC United States of America; 3 KHANA Center for Population Health Research, Phnom Penh, Cambodia; 4 Department of Preventive Medicine, Ministry of Health, Phnom Penh, Cambodia; 5 Case Western Reserve University, Cleveland, OH United States of America; 6 Center for Global Health Research, Touro University California, Vallejo, CA United States of America; 7 Saw Swee Hock School of Public Health, National University of Singapore, Singapore, Singapore

**Keywords:** Toolkits, Noncommunicable diseases, Primary care, Low- and middle-income countries, Asia

## Abstract

**Background:**

In Cambodia, economic development accompanied by health reforms has led to a rapidly ageing population and an increasing incidence and prevalence of noncommunicable diseases. National strategic plans recognize primary care health centres as the focal points of care for treating and managing chronic conditions, particularly hypertension and type 2 diabetes. However, health centres have limited experience in providing such services. This case study describes the process of developing a toolkit to facilitate the use of evidence-based guidelines to manage hypertension and type 2 diabetes at the health-centre level.

**Methods:**

We developed and revised a preliminary toolkit based on the feedback received from key stakeholders. We gathered feedback through an iterative process of group and one-to-one consultations with representatives of the Ministry of Health, provincial health department, health centres and nongovernmental organizations between April 2019 and March 2021.

**Results:**

A toolkit was developed and organized according to the core tasks required to treat and manage hypertension and type 2 diabetes patients. The main tools included patient identification and treatment cards, risk screening forms, a treatment flowchart, referral forms, and patient education material on risk factors and lifestyle recommendations on diet, exercise, and smoking cessation. The toolkit supplements existing guidelines by incorporating context-specific features, including drug availability and the types of medication and dosage guidelines recommended by the Ministry of Health. Referral forms can be extended to incorporate engagement with community health workers and patient education material adapted to the local context. All tools were translated into Khmer and can be modified as needed based on available resources and arrangements with other institutions.

**Conclusions:**

Our study demonstrates how a toolkit can be developed through iterative engagement with relevant stakeholders individually and in groups to support the implementation of evidence-based guidelines. Such toolkits can help strengthen the function and capacity of the primary care system to provide care for noncommunicable diseases, serving as the first step towards developing a more comprehensive and sustainable health system in the context of population ageing and caring for patients with chronic diseases.

**Supplementary Information:**

The online version contains supplementary material available at 10.1186/s12961-022-00912-4.

## Background

Populations worldwide are ageing rapidly, and accompanying this phenomenon is a rise in the prevalence of noncommunicable diseases (NCDs). Low- and middle-income countries (LMICs) report disproportionately higher disease and economic burden [1, 2]. In 2019, 74% of all deaths globally were caused by NCDs, and the proportion was 85% in LMICs in South-East Asia [3]. Most deaths from NCDs are attributable to cardiovascular diseases, followed by cancers, respiratory diseases, and type 2 diabetes.

In Cambodia, economic development accompanied by health reforms since the early 1990s has resulted in substantial life expectancy gains [4]. Falling fertility rates have followed other countries’ trajectories with a rapidly ageing population [5] and an increased prevalence of NCDs. The most common NCDs in Cambodia include type 2 diabetes, cancers, cardiovascular diseases, and chronic respiratory diseases [6].

During the past 15 years, the number and proportion of deaths due to NCDs in Cambodia has risen steeply, while total mortality has decreased. Mortality from NCDs has increased by more than 50%, from 38,600 deaths in 2000 to nearly 60,000 deaths in 2016 [3]. In 2016, 38% of deaths from NCDs were caused by cardiovascular diseases, 22% by cancers, 6% by respiratory diseases and 4% by type 2 diabetes [3]. The driving forces behind this epidemiological shift from communicable diseases to NCDs include rapid urbanization, unhealthy diets, lack of physical activity, and population ageing [2, 6].

The National Strategic Plan for the Prevention and Control of Noncommunicable Diseases 2013–2020 [7] outlines the adverse health and economic impacts of the increase in NCDs and the roles and responsibilities of the many government agencies enrolled to “break the cycle of poverty and non-communicable diseases”. The plan was supplemented by the 2018–2027 National Multisectoral Action Plan for the Prevention and Control of Noncommunicable Diseases [8]. In the near term, the strategic plan establishes primary care-level HCs as the focal points for NCD health services, specifically those related to managing hypertension and type 2 diabetes. However, HCs have limited experience in providing NCD services, primarily focusing on maternal and child health, communicable diseases, and health education and promotion services. Further, while evidence-based guidelines for managing common chronic conditions [9, 10] and efforts to guide HCs in establishing NCD services exist, such activities need to be tailored to the local context. One way to implement evidence-based guidelines is developing and applying a toolkit localized to a particular geographical or healthcare setting.

Toolkits can consist of one or a set of documents developed to support clinical workflows or pathways, targeting healthcare providers, community health workers, patients or other key stakeholders. Studies have shown that using a toolkit in conjunction with a clinical intervention is associated with improved health outcomes, including reduced falls, number of hyperglycaemic events, and lengths of stay [11].

This report documents the process of developing a toolkit designed to help physicians, nurses and other staff at HCs in Cambodia assess, counsel, treat and manage patients with hypertension and type 2 diabetes. While most engagement with stakeholders in this study has, for practical and financial reasons, focused only on Siem Reap Province, the tools may be adapted for use in other provinces in the country.

## Methods

### Funding

This research was supported by WHO’s Centre for Health Development. 

### Process of developing the toolkit

We developed the toolkit based on guidelines published by the WHO Package of Essential Noncommunicable Disease Interventions (WHO PEN) for primary healthcare [9]. In addition, we considered elements in the HEARTS technical package for cardiovascular disease management in primary healthcare [10] as they relate to promoting care processes through the use of standard tools. The work builds on engagement with stakeholders in Cambodia, which has occurred since 2016, examining the opportunities and challenges of offering enhanced NCD services [12].

The toolkit was developed using an iterative process of group and one-to-one consultations with key stakeholders facilitated by team members based in Cambodia. The stakeholders included representatives of the Ministry of Health (MOH), Siem Reap Provincial Health Department, national health institutes, HCs in all four operational districts of Siem Reap Province and nongovernmental organizations (NGOs). The consultations took place between April 2019 and March 2021. All one-to-one consultations with stakeholders were conducted directly in Khmer by Cambodian research team members. Group consultations were also conducted primarily in Khmer with a professional translator on-site to facilitate translations in English and Khmer in real time.

The development of the toolkit broadly followed the principles of facilitated process improvement, a methodology applied in other settings providing care for chronic diseases that explicitly takes account of context-specific features and challenges when developing a toolkit for translating evidence-based guidelines into practice [13]. This method followed four systematic steps:

1. Preliminary consultation with key stakeholders to broadly identify key bottlenecks in the prevention and management of NCD and tools to address them
2. Implementation of a capacity survey of HCs in Siem Reap province
3. Scoping review of recent primary care interventions for managing NCDs in LMICs
4. Development and assessment of the toolkit in a two-stage process with key stakeholders

These steps are summarized in Table 1 and described in greater detail in the following sections.

**Table 1:** Timeline of the development of a toolkit for evidence-based guidelines to treat hypertension and type 2 diabetes, Cambodia 2019–2021

Step	Purpose	Date
Step 1: consultative meeting with key stakeholders	• Identify key bottlenecks in the prevention and management of and screening for NCDs at the HC level in Siem Reap • Identify possible tools to address the bottlenecks • Review a questionnaire for assessing the capacity of HCs to provide primary NCD care for hypertension and type 2 diabetes	April 2019
Step 2: HC capacity survey	• Implement a cross-sectional facility survey among primary healthcare centres to assess the capacity of HCs in all four operational districts in Siem Reap to provide primary NCD care for hypertension and type 2 diabetes	June 2019
Step 3: scoping review	• Conduct a scoping review of recent primary care interventions for managing NCDs in low-resource settings in Asia	November 2019–March 2020
Step 4: development and assessment of the toolkit	• Develop a preliminary toolkit based on steps 1–3 • Introduce the preliminary toolkit to the stakeholder advisory group for feedback • Revise the toolkit based on the feedback and reassess it through semi-structured interviews with the Stakeholder Evaluation Group in Siem Reap and Phnom Penh • Finalize the toolkit based on the feedback from interviews with the evaluation group	April 2020–March 2021

NCDs: noncommunicable diseases

### Step 1: Consultative meeting with key stakeholders

**Fig. 1:**
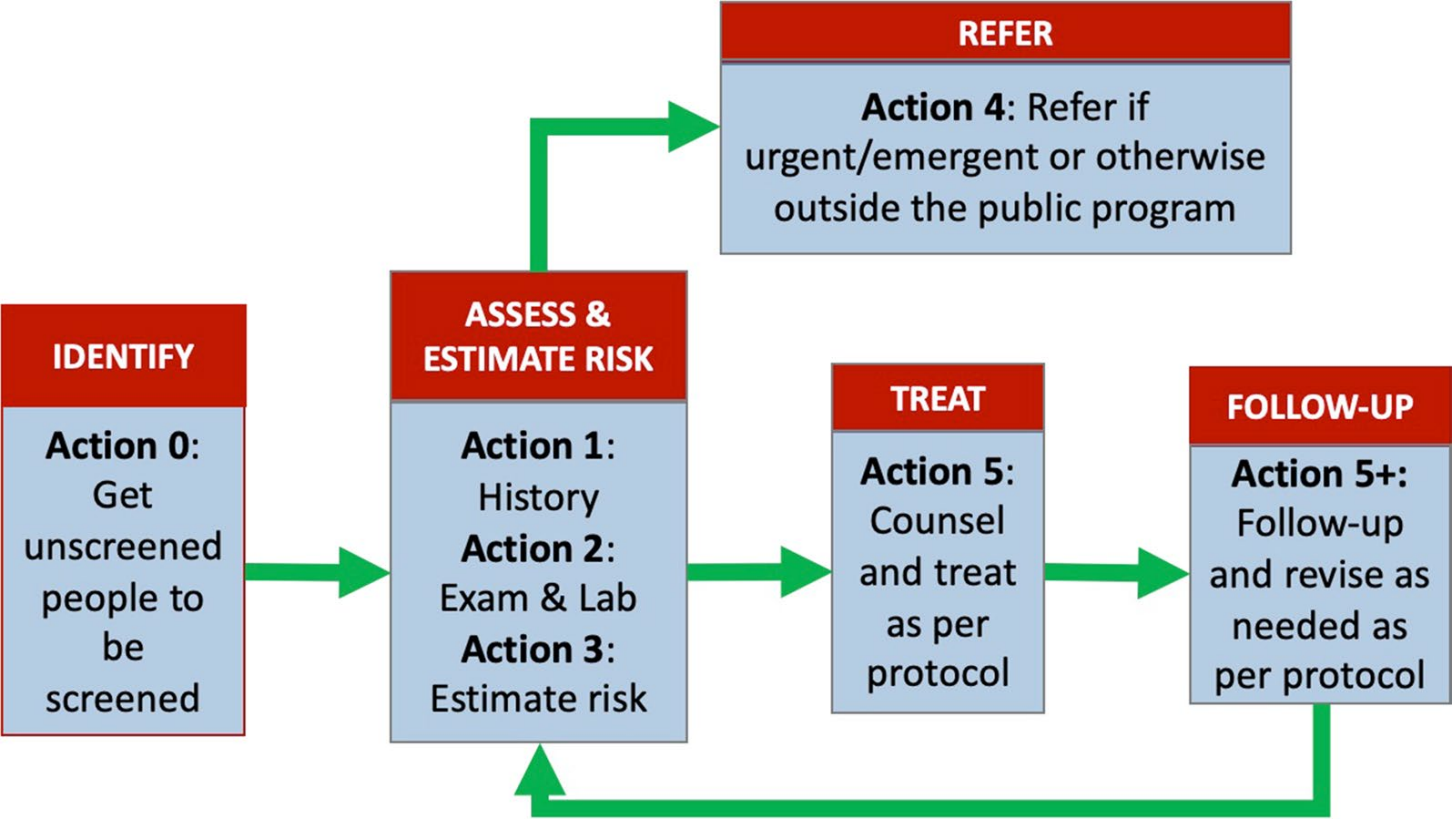
Core tasks in the management of hypertension and type 2 diabetes. Source: reference [15]

A 2-day meeting was held with stakeholders in April 2019 in Siem Reap, Cambodia. The consultative meeting aimed to identify the key bottlenecks to preventing, screening for and managing NCDs at the HC level in the province and the possible tools that could be used to address them, based on a set of core functional specifications for achieving WHO PEN (Fig. 1). The meeting comprised 30 participants, including representatives of the MOH, Siem Reap Provincial Health Department, operational districts and HCs in the province and NGOs working to improve NCD care in Cambodia.

Participants were first asked to identify a range of specific sites that could serve as archetypes for different HCs to better understand the challenges faced in providing NCD care in Cambodia. Three prototypical types were identified, based on actual HCs in Siem Reap, and these were used for further discussion.

During the workshop phase, a series of group exercises was conducted to outline and discuss a general representation of the main factors promoting or inhibiting successful management of NCDs, focusing on hypertension and type 2 diabetes. In addition, stakeholders provided feedback on a questionnaire developed for use to identify capacity gaps in providing NCD care across a range of HCs in the province.

The three thematic challenges to preventing and treating hypertension and type 2 diabetes were the accessibility and affordability of NCD services, awareness and knowledge of the benefits of NCD care, and the quality of care in HCs, particularly regarding the availability of medicines and appropriately trained staff. Subsequently, a list of measures related to structures, processes and outcomes was identified that could be used to track how well a HC performs in preventing and treating basic NCDs. The stakeholders also reviewed the draft survey designed to assess HCs’ capacities to provide NCD primary care.

### Step 2: Capacity survey among HCs

A cross-sectional facility survey was conducted in June 2019 among chiefs and vice-chiefs of primary healthcare centres to assess the capacity of the centres in Siem Reap Province to provide primary NCD care for hypertension and type 2 diabetes. The capacities assessed included the availability of equipment, provision of essential services, diagnostic capacity and the availability of essential medicines. The survey was conducted in all four operational districts in the province: Angkor Chum, Krolanh, Siem Reap and Sotr Nikum. Out of a total of 91 HCs in the province, a statistically representative sample of 45 was randomly selected, stratified by geographical location (urban, semiurban and rural) and the number of centres in each operational district. Data were collected using the WHO PEN sample questionnaire to assess capacity to prevent and manage major NCDs in primary care centres in low-resource settings [14]*.*

### Step 3: Scoping review of primary care interventions for managing NCDs

The next step involved a scoping review of recent primary care interventions for managing NCDs in LMICs in Asia. The review was initiated in November 2019 and conducted by two research team members. Articles were searched using the MEDLINE database. To be included, studies must have been published in peer-reviewed journals in English between 2014 and 2019, with interventions focusing on chronic conditions or NCDs (excluding mental health conditions and maternal care) or hypertension or type 2 diabetes (excluding gestational diabetes) or cardiovascular disease in primary healthcare settings in LMICs in Asia. We also included articles related to the management of NCDs and their associated complications, such as diabetic foot. Traditional and naturopathic medicine interventions and studies focused on allied health, specialist, and secondary or tertiary care were excluded.

A total of 191 articles were retrieved. Two reviewers independently screened the titles and abstracts, developed a list for inclusion and discussed discrepancies. Out of 191 articles found, 56 were selected for further review. Validity scores were given to assess the intervention’s acceptability, sustainability and impact on processes and control of the disease. Of the 56 articles, we identified 10 studies describing eight different trials as having adhered to most of the actionable steps associated with implementing a core package for managing hypertension or type 2 diabetes (Fig. 1). The resources described in these trials ranged from community screening to the diagnosis and management of patients.

### Step 4: Development and assessment of the toolkit

As part of the literature review, practical examples of the implementation of essential NCD interventions were identified, and the relevant means (tools) were extracted, if available. In addition, the team engaged with experts familiar with Cambodia’s health system infrastructure and who had experience conducting similar NCD programmes in Cambodia and other low-resource settings. Based on these findings, a draft list of tools was developed and presented and revised in a two-stage process.

The first stage consisted of a 1-day meeting with a stakeholder advisory group held in December 2020. The meeting brought together stakeholders from the MOH, Siem Reap Provincial Health Department, HCs, the Cambodia Diabetes Association, national health institutes and NGOs to assess the draft set of tools for implementing the core tasks of a basic package for managing and treating hypertension and type 2 diabetes. Participants were requested to critique the tools in terms of their sufficiency and practicality for implementation at the same prototypical sites of care discussed earlier. Suggested improvements were sought for any tools deemed to be less than sufficient or practical, and the tools were revised accordingly. Findings from this meeting fed into the development of the first complete version of the toolkit, and they were used to describe each tool’s purpose and intended users and provide a brief explanation of how to use it.

The second stage involved presenting the revised toolkit to a stakeholder evaluation group and assessing it through a series of 10 one-on-one interviews with the group members, all of whom had direct experience in providing services, administering or overseeing potential WHO PEN sites in the province. Feedback from these interviews was incorporated into the final toolkit described in the Results section.

## Results

The preliminary toolkit was developed, critiqued and revised based on systematic consultations with key stakeholders. The toolkit aims to help physicians, nurses and other staff at primary care-level HCs in Cambodia assess, counsel, treat and manage patients with hypertension and type 2 diabetes. Based on evidence-based guidelines, the toolkit can be used for the routine management of patients with elevated blood pressure or blood glucose and to identify potential patients, such as those with a history of cardiovascular disease in first-degree relatives, those who are smokers or obese and those aged ≥ 40 years.

The toolkit—known as *A toolkit for hypertension and diabetes management at health centres in Cambodia* [15]—is organized according to the core tasks required to treat and manage these diseases, as summarized in Fig. 1. A supporting set of administrative tools is included in the appendix of the toolkit to facilitate resource management and quality improvement. A full version of the toolkit is included in Additional file 1: Appendix 1.

Table 2 summarizes the tools, the purpose of each tool and its intended users. This toolkit is implemented in a paper version, and the descriptions specify where each document should be kept. Each tool will be filed in either a clinic registration file or a patient’s record or held by the patient. The patient’s record can be subdivided into administrative, care encounters (including missed visits and referral consultations) and laboratory records.

**Table 2:** Tools in *A toolkit for hypertension and diabetes management at health centres in Cambodia* (action numbers correspond to those in Fig. 1)

Action number	Tool number	Tool name	Intended user	Tool description
Action 0: Get unscreened people to be screened	0.1	Patient identification (ID) card	Clinic staff	This card is to be issued by clinic staff for new visitors to the HC and held by the patient. The ID card will have a unique HC ID issued to each patient by the HC, along with other personal and contact information. The patient ID card would, ideally, be linked to a national ID such as their Patient Management and Registration System or patient ID if available, along with information such as the health insurance/financing scheme they belong to or any means-tested benefits the patient may be eligible for at the time of payment
	0.2	Risk-screening form	Nurse, clinic staff	This form is to be filled in by clinic staff for all adult patients at their first visit and, subsequently, every 5 years The information collected in this form identifies whether a patient is a potential user of services aimed at treating type 2 diabetes and/or hypertension as indicated in the *treatment algorithm (Tool 1.1)*. If they are, the attending nurse or physician shall proceed with filling in the *treatment card (Tool 1.2)* under the column “intake visit”, either on the day itself or at a follow-up confirmatory visit For HCs collaborating with CHWs such as a PE or VHSG, this form can be used as a community screening form. Here, the form would be filled by the CHW, with a copy of this form retained by the CHW and a duplicate copy shared with the HC. The HC and CHW would then coordinate to arrange follow-up visits at the HC or community level
Action 1: History-taking	1.1	Treatment algorithm	Nurse, physician	The algorithm guides the attending nurse or physician to a treatment plan tailored to individuals based on their personal and familial history of CVD and assesses their lifestyle risk factors. The healthcare provider will use the algorithm to determine whether the patient should be managed locally or referred to a higher-level facility. If managed locally, the algorithm guides the nurse or physician in managing the patient at the clinic using the information recorded in the *treatment card (Tool 1.2)*. The algorithm is designed based on the MOH’s National Standard Operating Procedure for Diabetes and Hypertension Management in Primary Care in Cambodia [16]
	1.2	Treatment card	Nurse, physician	This card is to be filled in by the healthcare provider to whom the patient has been referred for evaluation The card documents essential information for monitoring treatment of CVD risk and tracking changes in health and treatment. The form is the place to record key data the provider needs to assess CVD risk using the *risk-based charts (Tool 3)* and follows the *treatment algorithm (Tool 1.1)*
Action 2: Exams and lab tests	2	Laboratory flowsheet	Nurse, physician	The *laboratory flowsheet* is to be filled in by the healthcare provider based on point-of-care testing or outside laboratory results. The data elements are focused on the information required to follow the *treatment algorithm (Tool 1.1)* for managing elevated blood pressure The flowsheet is to be used for HCs that have access to outside or more advanced lab testing
Action 3: Estimate risk	3	Risk-based charts for hypertension	Nurse, physician	The *risk-based charts* are CVD risk non-laboratory-based charts published in the WHO’s HEARTS package [10] and translated into Khmer for local use at the HC
Action 4: Refer	4	Referral form (to referral institution)	Nurse, physician	The referral institution (RI) is a designated centre for receiving hypertensive or diabetic patients deemed by the algorithm to require more advanced evaluations, either a clinic or a hospital. Alternatively, the HC may have a special clinic located at the HC to take NCD patients on designated days The attending nurse or physician filled in the *referral form* to refer patients to a higher-level facility as indicated in the *treatment algorithm (Tool 1.1)* The intended use of this form is to facilitate appointments made at the HC for a visit to a RI. Ideally, the RI would receive a copy of the referral form in advance of the patient’s visit and would, likewise, send a copy of the feedback form back to the HC in advance of the patient’s follow-up visit at the HC. If this is not possible, a modification will need to be made to simplify the process
Action 5: Counsel and treat as per protocol	5.1	Patient education material	Nurse, physician	The attending nurse or physician can use the patient education material to counsel all patients on risk factors for CVD and how to recognize symptoms. The material also includes information on how lifestyle changes in diet, exercise and smoking can be used to lower one’s risk of developing CVD NCD awareness and prevention posters can be taped to a consultation wall or showcased at visible places at the HC. NCD leaflets can also be shared with patients to take home if sufficient resources are available
	5.2	Prescription form (general purpose)	Nurse, physician	The *prescription form* is to be filled in by the attending nurse or physician to prescribe medications to patients visiting the HC. The form can be customized based on medications recommended in the *treatment algorithm (Tool 1.1)* and/or available at the HC. The healthcare provider can also fill in the form to purchase medications outside the HC, such as a private pharmacy, if unavailable in house. The prescription form will be handed to and retained by the pharmacist and a receipt issued to the patient on receipt of payment Drugs included in the form would have to be consistent with those locally available either through the CMS or at a private pharmacy
Action 6: Follow up and revise as needed as per protocol	6.1	Patient missed visit form	Nurse, clinic staff	The *patient missed visit form* is to be filled in by clinic staff to record missed visits at the HC. It can also be used to keep track of rescheduled visits. The form assumes that contact can be made via a phone call; this can be modified to accommodate other means of contact, including text messages or house visits
	6.2	Patient exit form	Clinic staff	The *patient exit form* is to be filled in by clinic staff to de-register patients at the HC for various reasons, including death, LTFU or patients declining care. If LTFU, the HC shall determine an appropriate definition to assign to the same

*Source* Reference [15]

*CHW* community health worker; *CMS* central medical store; *CVD* cardiovascular disease; *HC* health centre; *ID* identification card; *LTFU* lost to follow-up; *MOH* Ministry of Health; *PE* peer educator; *RI* referral institution; *VHSG* village health support group

The toolkit was designed to be practical and straightforward to follow and supplement the existing evidence-based guidelines [9, 10] that promote care processes using standard tools.

The toolkit can be modified as needed based on the resources at HCs, operational districts or provinces, or arrangements with other institutions, such as referral facilities, networks of community health workers, private pharmacies, general practitioners and NGOs.

Seven assumptions were made when developing the toolkit.

1. The HC is the focal point for care for hypertension and type 2 diabetes, and it has clear and effective links to referral institutions.
2. Tools for community outreach will be developed only after a solid relationship between the HC and referral institution is established.
3. Medications are assumed to be available and affordable.
4. The tools should focus on best addressing the task function and should not necessarily restrict how and by whom the tasks are performed.
5. The tools address essential tasks and include the key information and procedures required for fulfilling the functional specifications. These tools can be expanded to address needs specific to each HC, operational district or province.
6. The typical HC that would benefit most from the toolkit would be one functioning relatively well in its traditional roles, does not need the involvement of community health workers and has demonstrated administrative capacity in carrying out day-to-day activities.
7. Implementing the toolkit also involves a training manual and consulting assistance, the details of which are not described here.

## Discussion

The Toolkit for Hypertension and Diabetes Management at HCs in Cambodia was developed to help healthcare providers implement established evidence-based guidelines on managing hypertension and type 2 diabetes at the primary care level.

The development of the toolkit was undertaken as an iterative process through engagement with stakeholders at multiple intervals. This engagement allowed us to better tailor the tools to the local context while also strengthening buy-in at the ministry and HC level in need of such a toolkit for facilitating the implementation of evidence-based guidelines at the local level. This case study has also shown that research teams from multiple countries, including LMICs, can collectively work together to implement a project that incorporates international and context-specific features and produce a document that can be implemented at primary care facilities. Finally, the toolkit has been developed so that the majority of the tools can be translated and implemented as-is in other primary care facilities in other provinces in Cambodia and other LMICs.

This case study has a few limitations. First, given that this project focused on HCs in Siem Reap Province, home to the second-largest city in the country, the results may not be generalizable to other provinces whose healthcare infrastructure is different and where geographical proximity to healthcare facilities may vary. Second, given the set of assumptions made in the toolkit development, successful deployment will require other inter-related factors to be addressed. Two main challenges in this regard are the reliable procurement and supply chain management of medication and the referral systems in place between different sites of care. It is crucial to ensure medication availability and affordability and improve the integration of HCs with and referral systems between HCs and higher-level facilities, such as referral hospitals, for the functional specifications highlighted in the WHO PEN to be successfully implemented. Third, stakeholder consultations did not include patients, the indirect (but ultimate) beneficiaries of the toolkit. Patient-level input is highly valuable in any implementation science project. We intend to seek and incorporate patient-level feedback in iterations of the toolkit in follow-up research studies. Fourth, while contextual features were incorporated into the final toolkit, a formal contextual assessment was not conducted due to time and resource constraints. Fifth, since the completion of the literature review and development of the toolkit, two sets of related international guidelines have since been published, namely, guidelines for the management of hypertension with a special focus on LMICs published by the International Society of Hypertension in 2020, and pharmacological guidelines for hypertension management published by WHO in 2021; these documents are important to take note of and will be evaluated and reviewed in future iterations of the toolkit. Finally, user manuals and training curricula should be developed to support their understanding and use for the toolkits to be implemented as intended. Future projects aim to develop a user manual and a training package to support healthcare workers in implementing the toolkit and pilot test the toolkit and training package in selected sites in Siem Reap Province.

The toolkit is a work in progress, and the team envisages that changes will be made as it is pilot tested in a few sites, outside the scope of this project. Steps will also be taken to harmonize information captured in the toolkit with an emerging electronic NCD database being developed by the MOH. While the tools are presented as paper-based, they are designed to be translated into an electronic medical record.

It is envisaged that strengthening primary care at the HC level will serve as the first step towards developing a more comprehensive system in which workers at the community level and physicians at higher-level facilities, such as NCD clinics and hospitals, will be incorporated into the care pathway for NCD patients. The resources appropriate for this more extensive ecosystem would be incorporated into version 2.0 of the toolkit.

## Conclusions

Toolkits can support the implementation of evidence-based guidelines. A toolkit was developed based on systematic consultations with key stakeholders to help staff at primary HCs in Cambodia assess, counsel, treat and manage patients with hypertension and type 2 diabetes. The toolkit can be modified based on the available resources and arrangements with other institutions. These tools are designed to be practical and specific enough to be implemented in the near term in local sites, and there are plans to expand nationally in the medium to long term.

Implementation of this toolkit can assist health workers, from the community level to physicians at tertiary facilities, in strengthening the functioning and capacity of the primary care system to provide NCD care. It also serves as the first step towards developing a more comprehensive and sustainable health system in the context of population ageing.

## Supplementary Information

**Additional file 1: Appendix S1.** A toolkit for hypertension and diabetes management at health centres in Cambodia is the final toolkit developed as a result of the process described in this paper. It contains a set of tools developed to assist healthcare workers responsible for diabetes and hypertension care management at health centres to improve the effectiveness and quality of services provided.

## Data Availability

The datasets are available from the corresponding author on reasonable request.

## Abbreviations

CHW: Community health worker
CMS: Central medical store
CVD: Cardiovascular disease
HC: Health centre
ID: Identification card
LTFU: Lost to follow-up
MOH: Ministry of Health
NCD: Noncommunicable disease
NGO: Nongovernmental organization
PE: Peer educator
RI: Referral institution
VHSG: Village health support group
WHO PEN: WHO Package of Essential Noncommunicable Disease Interventions

## References

[1] Abegunde DO (2007). The burden and costs of chronic diseases in low-income and middle-income countries. Lancet.

[2] Boutayeb A, Boutayeb S (2005). The burden of non communicable diseases in developing countries. Int J Equity Health.

[3] World Health Organization (2020). Global health estimates 2020: deaths by cause, age, sex, by country and by region, 2000–2019, in health: risk factors.

[4] Annear PL, Jacobs B, Nachtnebel M (2015). The Kingdom of Cambodia health system review. Health systems in transition.

[5] National Institute of Statistics/Cambodia, Directorate General for Health/Cambodia, and ICF International, *Cambodia Demographic and Health Survey 2014*. 2015, National Institute of Statistics/Cambodia, Directorate General for Health/Cambodia, and ICF International: Phnom Penh, Cambodia.

[6] Oum S, et al., *Prevalence of Non-Communicable Disease Risk Factors in Cambodia: STEPS Survey Country Report*. 2010, University of Health Sciences: Phnom Penh, Cambodia.

[7] Ministry of Health, *National Strategic Plan for the Prevention and Control of Noncommunicable Diseases: 2013–2020*. 2013, Ministry of Health: Phnom Penh, Cambodia.

[8] Ministry of Health, *National Multisectoral Action Plan for the Prevention and Control of Noncommunicable Diseases*. 2018, Ministry of Health: Phnom Penh, Cambodia.

[9] World Health Organization (2020). WHO package of essential noncommunicable (PEN) disease interventions for primary health care.

[10] World Health Organization (2018). HEARTS Technical package for cardiovascular disease management in primary health care: implementation guide.

[11] Thoele K (2020). Development and use of a toolkit to facilitate implementation of an evidence-based intervention: a descriptive case study. Implement Sci Commun.

[12] Ansah JP (2019). Systems modelling as an approach for understanding and building consensus on non-communicable diseases (NCD) management in Cambodia. BMC Health Serv Res.

[13] Matchar DB (2006). Facilitated process improvement: an approach to the seamless linkage between evidence and practice in CKD. Am J Kidney Dis.

[14] World Health Organization (2010). Package of essential noncommunicable (PEN) disease interventions for primary health care in low-resource settings.

[15] Department of Preventive Medicine, M.o.H., *A toolkit for hypertension and diabetes management at health centres in Cambodia*. 2021, Ministry of Health: Phnom Penh.

[16] Ministry of Health, *National Standard Operating Procedure for Diabetes and Hypertension Management in Primary Care*, D.o.P. Medicine, Editor. 2019, Ministry of Health: Phnom Penh.

