# Supplementary material for: Developing a toolkit for implementing evidence-based guidelines to manage hypertension and diabetes in Cambodia: a descriptive case study
Source: Health Res Policy Syst. 2022 Nov 29;20(Suppl 1):109. doi: 10.1186/s12961-022-00912-4 (PMC9706829; doi:10.1186/s12961-022-00912-4)
Supplement: Supplementary file 1 — Additional file 1: Appendix S1. A toolkit for hypertension and diabetes management at health centres in Cambodia is the final toolkit developed as a result of the process described in this paper. It contains a set of tools developed to assist healthcare workers responsible for diabetes and hypertension care management at health centres to improve the effectiveness and quality of services provided. [file 12961_2022_912_MOESM1_ESM.docx]

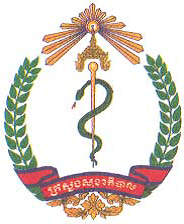


A Toolkit for Hypertension and Diabetes Management at Health Centres in Cambodia


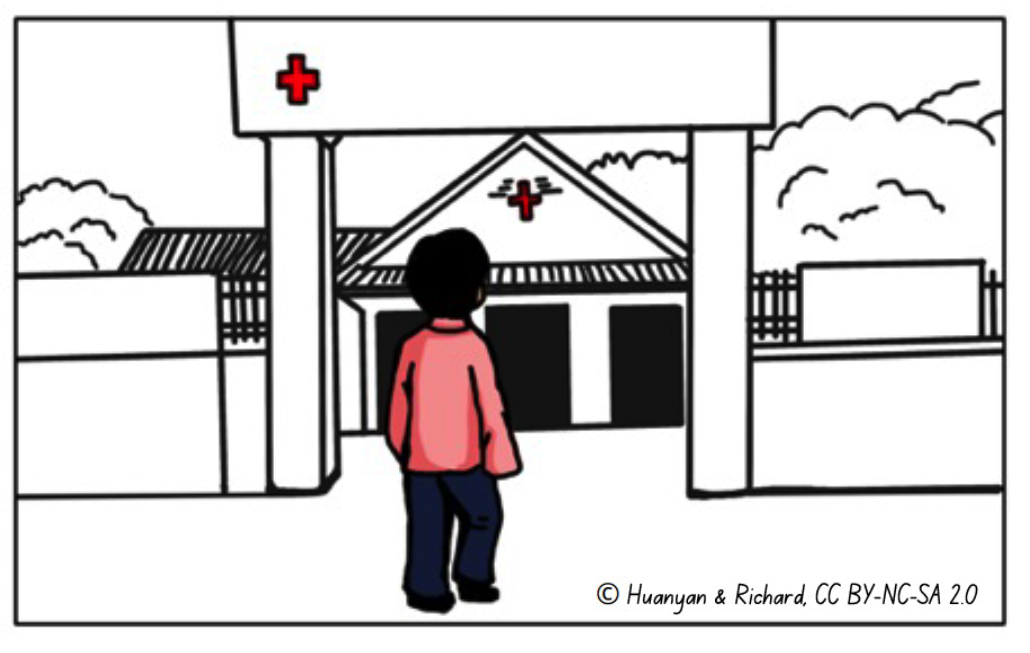


Department of Preventive Medicine, Ministry of Health

May 2021

Contents

[List of Figures 5](#_Toc98486444)

[List of Tables 6](#_Toc98486445)

[Preface 7](#_Toc98486446)

[Acknowledgements 8](#_Toc98486447)

[Team Members 8](#_Toc98486448)

[List of Abbreviations 9](#_Toc98486449)

[Executive Summary 10](#_Toc98486450)

[1. Introduction 11](#_Toc98486451)

[0.1. Patient ID card 18](#_Toc98486452)

[0.2. Risk screening form 20](#_Toc98486453)

[1.1. Treatment algorithm (MOH Cambodia) 22](#_Toc98486454)

[1.2 Treatment card (facility) 24](#_Toc98486455)

[2. Laboratory flowsheet 29](#_Toc98486456)

[3. Risk-based charts 31](#_Toc98486457)

[4. Referral form (referral institution) 33](#_Toc98486458)

[5. Counsel and Treat 35](#_Toc98486459)

[5.1 Patient education material 35](#_Toc98486460)

[5.2. Prescription form (general purpose) 42](#_Toc98486461)

[6. Forms for missed visits 43](#_Toc98486462)

[6.1. Patient missed visit form 43](#_Toc98486463)

[6.2. Patient exit form 44](#_Toc98486464)

[Appendix 45](#_Toc98486465)

[A1. Referral form (community health worker) 46](#_Toc98486466)

[A2. Employee sign-in sheet 48](#_Toc98486467)

[A3. Stock card 49](#_Toc98486468)

[A4. Technology needs assessment 50](#_Toc98486469)

[A5. Medicines needs assessment 52](#_Toc98486470)

[A6. Purchase order/voucher 53](#_Toc98486471)

[A7. Budget sheet 54](#_Toc98486472)

[A8. Cashflow projection sheet 55](#_Toc98486473)

[A9. Facility register for monitoring outcomes 56](#_Toc98486474)

[A10. Health facility report 57](#_Toc98486475)

[A11. Patient report card 59](#_Toc98486476)

[A12. Treatment supervision/audit form 60](#_Toc98486477)

[A13. Summary of supervision visits 65](#_Toc98486478)

[A14. Pre-visit planning: Workflow mapping worksheet 66](#_Toc98486479)

[A15. Pre-visit planning: Assessment of current practice 67](#_Toc98486480)

[A16. Patient visit: Workflow mapping worksheet 68](#_Toc98486481)

[A17. Patient visit: Assessment of current practice 71](#_Toc98486482)

[A18. Post-visit follow-up: Workflow mapping sheet 74](#_Toc98486483)

[A19. Post-visit follow-up: Assessment of current practice 75](#_Toc98486484)

[References 76](#_Toc98486485)

# List of Figures

[**Figure 1: Deaths by cause (in thousands) 11**](#_Toc98486361)

[**Figure 2: WHO PEN core tasks 15**](#_Toc98486362)

[**Figure 3: Patient flow pathway 17**](#_Toc98486363)

# List of Tables

[**Table 1: Prevalence of NCD Risk Factors 12**](#_Toc89679374)

[**Table 2: List of tools 16**](#_Toc89679375)

# Preface

The Toolkit for Hypertension and Diabetes Management at Health Centres in Cambodia has been developed to assist healthcare workers, responsible for diabetes and hypertension care management at health centers in improving the effectiveness and quality of services provided.

This package illustrates the immense effort of the Ministry of Health (MOH) in addressing the growing epidemiological and financial challenges of non-communicable diseases (NCDs). The common set of lifestyle factors contributing to the rise in the incidence and prevalence of diabetes and hypertension include unhealthy diets, physical inactivity, obesity, excessive alcohol consumption and smoking. This could eventually lead to serious complications such as blindness, kidney damage, leg ulcers, and other disabilities, and premature mortality.

In line with the vision and goals highlighted in the National Health Strategic Plan for Prevention and Control of Non-communicable Diseases (2013-2020) and 2030 UN Sustainable Development Goals in Cambodia, the MOH is confident that the use of this Toolkit can improve the quality and accountability of NCD health care services provided at the outpatient level.

# Acknowledgements

We would like to thank the professors, doctors, pharmacists, health officers at both national and sub-national levels and the rest of the team who spent their valuable time in developing, reviewing, and editing the Toolkit for Hypertension and Diabetes Management at Health Centres in Cambodia to successful completion. This research was supported by the World Health Organisation Centre for Health Development (WHO Kobe Centre – WKC: K18018).

#

# Team Members

|  | **Dr. Kol Hero** | Director, Preventive Medicine Department | Ministry of Health, Cambodia |
| --- | --- | --- | --- |
|  | **Prof. David Matchar** | Professor, Program in Health Services and Systems Research | Duke-NUS Medical School, Singapore |
|  | **A/Prof. Dr. Yi Siyan** | Director, KHANA Center for Population Health Research | KHANA, Cambodia |
|  | **Dr. Chhun Lun** | Chief, NCD Bureau, Preventive Medicine Department | Ministry of Health, Cambodia |
|  | **Ms. Nirmali Sivapragasam** | Senior Research Associate, Program in Health Services and Systems Research | Duke-NUS Medical School, Singapore |
|  | **Mr. Chhoun Pheak** | Research Fellow, KHANA Center for Population Health Research | KHANA, Cambodia |
|  | **Dr. Sok Kung** | Deputy Chief, NCD Bureau, Preventive Medicine Department | Ministry of Health, Cambodia |
|  | **Mr. Tuot Sovannary** | Research Manager, KHANA Center for Population Health Research | KHANA, Cambodia |
|  | **A/Prof. John Ansah** | Assistant Professor, Program in Health Services and Systems Research | Duke-NUS Medical School, Singapore |
|  | **Ms. Amina Islam** | Deputy Director, Program Development and Business Administration, SingHealth Duke-NUS Global Health Institute | Duke-NUS Medical School, Singapore |
|  | **Ms. Tessa Lui** | Research Assistant, Program in Health Services and Systems Research | Duke-NUS Medical School, Singapore |
|  | **Ms. Xin Ya Lim** | MD Student | Duke-NUS Medical School, Singapore |
|  | **Dr. Muy Seanghorn** | Deputy Director, Preventive Medicine Department | Ministry of Health, Cambodia |
|  | **Dr. Seng Ratana** | Deputy Chief, NCD Control Office | Ministry of Health, Cambodia |
|  | **Dr. Phy Maly** | Officer, NCD Control Office | Ministry of Health, Cambodia |

#

# List of Abbreviations

| 12M | 12 months |
| --- | --- |
| 1M | 1 month |
| 6M | 6 months |
| BP | Blood pressure |
| CHW | Community health worker |
| CMS | Central Medical Store |
| CVD | Cardiovascular disease |
| DM | Diabetes Mellitus |
| FBG | Fasting blood glucose |
| FU | Follow-up |
| GP | General practitioner |
| HbA1c | Haemoglobin A1c |
| HC | Health centre |
| HCTZ | Hydrochlorothiazide |
| HDL | High-density lipoprotein |
| HT | Hypertension |
| LDL | Low-density lipoprotein |
| LTFU | Lost-to-follow-up |
| MOH | Ministry of Health |
| NCD | Noncommunicable disease |
| NGO | Non-governmental organization |
| PE | Peer educator |
| PHD | Provincial Health Department |
| PMRS | Patient Management and Registration System |
| RBG | Random blood glucose |
| RI | Referral institution |
| VHSG | Village health support group |
| WHO | World Health Organization |
| WHO PEN | WHO Package of Essential Noncommunicable Disease Interventions |

# Executive Summary

Economic development, together with health reforms since the early 1990s, have resulted in substantial gains in life expectancy in Cambodia (Annear et al., 2015). However, while total mortality has decreased during the period over the last 15 years, the number as well as the proportion of deaths due to non-communicable diseases (NCDs) has risen steeply.

The National Strategic Plan for the Prevention and Control of Non-communicable Diseases 2013-2020 (MoH, 2013) and the National Multisectoral action plan for the Prevention and Control of Noncommunicable Diseases 2018-2027 (MoH, 2018) highlights the WHO Package of Essential NCD Interventions (WHO PEN) as a set of interventions that can be implemented in low-resource settings such as Cambodia. The Plans also establish primary-care level health centres (HCs) as the focal point for health services related to the PEN, specifically hypertension (HT) and diabetes mellitus (DM) management in the near term. However, at present, HCs have limited experience in providing NCD services, with the majority of care and HCs visits being related to maternal and child health (MCH), communicable disease and basic health education and promotion services.

While there have been efforts at providing guidance to HCs in the establishment of NCD health services, such activities need to be tailored to the local context. Working with existing efforts, it is against this backdrop that we’ve identified a set of tools for implementing the WHO PEN and tailored these tools to the local context of HCs in Cambodia. This effort has been developed by Duke-NUS Medical School in collaboration with the Department of Preventive Medicine, Ministry of Health (MOH), Cambodia, and KHANA Centre for Population Health Research, Cambodia.

The intention of these tools is that they are practical and specific enough to be implemented in the near term in local sites with plans to expand it nationally in the medium to long term.

# 1. Introduction

Economic development, together with health reforms since the early 1990s, have resulted in substantial gains in life expectancy in Cambodia (Annear et al., 2015). According to latest estimates from Cambodia, infant and under-five mortality rates were 18 and 28 per 1000 live births, respectively, while the maternal mortality rate was 141 per 100,000 live births. Further, total fertility rate has declined to 2.4 children per woman while the average life expectancy has increased to 75.5 years (76.8 years for women and 74.3 years for men) (NIS, 2020).

Lower death rates together with falling fertility rates – the so-called ‘demographic transition’ – follows other countries’ trajectories, resulting in a rapidly ageing population (NIS, 2015), changes that have been highlighted in the Cambodia’s National Policy on Aging 2017-2030 (MoSVY, 2017).

One consequence of these demographic realities is that Cambodia is faced with the challenge of an increasing prevalence of non-communicable diseases (NCDs) and their risk factors. Major NCDs in Cambodia include diabetes mellitus (DM), cancer, cardiovascular diseases and chronic respiratory diseases (UHS, 2010).

**Figure 1: Deaths by cause (in thousands)**

Further, while total mortality has decreased during the period over the last 15 years, the number as well as the proportion of deaths due to NCDs has risen steeply. Mortality from NCDs has increased by more than 50% over the last 15 years from 38,600 deaths in 2000 to nearly 60,000 deaths in 2016 (WHO, 2018). Cardiovascular diseases and cancers accounted for 38% and 22% of total NCD deaths, respectively, while respiratory diseases and DM accounted for another 6% and 4% of total NCD deaths in 2016 (Figure 1) (WHO, 2018).

The common set of lifestyle factors contributing to the rise in NCDs is the triad of unhealthy diets, physical inactivity and tobacco use. Other harmful exposures include the excessive use of alcohol and indoor air pollution. According to the 2010 Cambodia STEPS (WHO STEPwise approach to Surveillance) survey, a nationally representative survey on prevalence of NCD risk factors, 15% of the population is overweight, 16% have hypertension, 1 in 5 have raised total cholesterol, and tobacco use is estimated at 37% (UHS, 2010). While there is a decrease in the prevalence of hypertension (14.5%) and the number of smokers (21.3%), most of these figures rise significantly based on STEPs 2016 as 21.9% of the population between the ages of 25-64 is overweight, 48.4% have raised total cholesterol, with 1.5% prevalence of diabetes (UHS, 2017). Alarmingly, as the population ages and becomes more urban, the prevalence of key risk factors increases (Table 1). Given the increase in the prevalence of risk factors identified earlier, these causes of morbidity and mortality are projected to rise if specific interventions to control risk factors are not implemented.​

**Table 1: Prevalence of NCD Risk Factors based on 2010 STEPs**

| **Prevalence of risk factors** | **Prevalence of risk factors by age group and location** |
| --- | --- |
| Overweight 15% | higher in urban than in rural (27% vs 13%)  increased with age (10% in 25-34 vs 18% in 55-64) |
| Hypertension 16% | higher in urban than in rural (26% vs 14%)  increased with age (6% in 25-34 vs 37% in 55-64) |
| Raised Total Cholesterol 21% | higher in urban than in rural (40% vs 21%)  increased with age (16% in 25-34 vs 38% in 55-64) |
| Tobacco 37% | higher in rural than in urban (40% vs 25%)  increased with age (26% in 25-34 vs 53% in 55-64) |

The National Strategic Plan for the Prevention and Control of Non-communicable Diseases 2013-2020 (MoH, 2013) outlines the negative impact of the increase in NCDs on the health system and the wellbeing of the people, as well as the economic impact due to lost productivity. The current strategy is built on the premise of adopting a “coherent and integrated” approach to achieve its objective of an equitable, cost effective and responsive health system (MoH, 2013) Early detection, preventative programs and better management are seen as the main pathways to manage the increasing number of NCD patients (MoH, 2013).

The Strategic Plan is further supplemented by the 2018-2027 National Multisectoral action plan for the Prevention and Control of Noncommunicable Diseases (MoH, 2018), which outlines roles and responsibilities of the many government agencies that have been enrolled in the effort to “break the cycle of poverty and noncommunicable diseases”. The multisectoral action plans recognises the benefits of adopting a coordinated approach; implementing complementary activities that address the underlying causes of diseases allows wins for all and negates issues of competing priorities. Such an approach minimises duplication of efforts and maximises the use of resources. RGC has identified a reduction in the risk factors leading to the four diseases (CVD, cancer, chronic respiratory disease and DM) as the main focus of interventions. The multisectoral action plan calls for “*effective screening, treatment and palliative care and … multisectoral collaboration to address other causes.”*

However, at present, HCs have limited experience in providing NCD services, with the majority of care related to maternal and child health (MCH), communicable disease and basic health education and promotion services. Further, while there have been efforts at providing guidance to HCs in the establishment of NCD health services, such activities need to be tailored to the local context.

Working with existing efforts, it is against this backdrop that we’ve identified a set of tools for implementing the WHO PEN and tailored these tools to the local context of HCs in Cambodia.

**The Toolkit for Hypertension and Diabetes Management at Health Centres in Cambodia** (hereafter referred to as The Toolkit) has been developed to help physicians, nurses and other staff at primary care-level HCs in Cambodia assess, counsel, treat and manage patients with HT and DM. This effort has been coordinated by Duke-NUS Medical School in collaboration with the Department of Preventive Medicine, Ministry of Health (MOH), Cambodia, and KHANA Center for Population Health Research, Cambodia.

The Toolkit is designed to be simple and practical to follow and supplements evidence-based guidelines published in the WHO Package of Essential Noncommunicable (PEN) Disease Interventions for Primary Health Care (WHO, 2020). In addition, we took into consideration elements in the HEARTS technical packages (WHO, 2018) as they relate to promoting care processes through the use of standard tools.

The tools were developed and evaluated through stakeholder meetings with the MOH, the Provincial Health Department (PHD) in Siem Reap, representatives of HCs and operational districts in the Siem Reap province and non-profit organisations supplemented by extensive literature reviews. Users of the tools are free to reproduce as is or modify the tools to suit their practice needs or preferences.

As indicated in the WHO PEN, the tools can be used for routine management of patients with known elevated blood pressure (BP) or blood glucose as well as identify potential patients at a higher risk of developing the same such as those with a history CVD in first-degree relatives, smokers, the obese and those aged 40 years and above.

A few assumptions were made when developing the Toolkit:

1. The HC is the focal point for HT and DM care, with a clear and effective link to the referral institution.
2. Tools for linking to communities will be considered only after a solid relationship between the HC and referral institution are established.
3. Medications are assumed to be available and affordable.
4. The tools should not restrict how the tasks are performed, and by whom. The tools focus on how to best address the task function.
5. The tools address essential tasks and include only key information and procedures required for fulfilling the PEN package. These tools can be expanded to address needs specific to each HC / district / province.
6. The typical HC that would benefit most from the Toolkit would be one that is functioning relatively well in its traditional roles of maternal and child health (MCH), communicable disease and basic health education and promotion services without the need for community health worker (CHW)^[[1]](#footnote-2)^ involvement and has demonstrated administrative capacity in carrying out day-to-day activities.
7. Implementing the Toolkit will also involve a training manual and consulting assistance, the details of which are not described here.

The Toolkit can be modified as needed based on the resources within the HC /district / province and arrangements in place with other institutions such as referral facilities, CHW networks (such as peer educators [PE] or village health support groups [VHSGs]), private pharmacies or general practitioners (GPs) and non-governmental organisations (NGOs).

It is envisaged that strengthening primary care at the HC level will serve as the first step to a more comprehensive system where workers at the community level as well as physicians at higher-level facilities such as at NCD clinics and hospitals will be incorporated in the care flow pathway for NCD patients; resources appropriate for this more extensive ecosystem would be incorporated into a version “2.0” of Toolkit.

It is noted that information captured in the Toolkit would need to harmonised with an emerging electronic NCD database being developed by the MOH before the Toolkit can be piloted in local sites. While the tools are presented as paper-based, they were designed in a way that can be translated into an electronic medical record.

The Toolkit is organized according to core tasks required to implement the PEN (Figure 2). A supporting set of administrative tools are also included in the Appendix to facilitate resource management and quality improvement.

Table 2 provides a summary of the tools, purpose of each tool and intended user. As this Toolkit is implemented here in a paper version, the tool descriptions also specify where each document should be kept^[[2]](#footnote-3)^. Figure 3 also illustrates how each tool fits into the general patient flow pathway for providing NCD care.


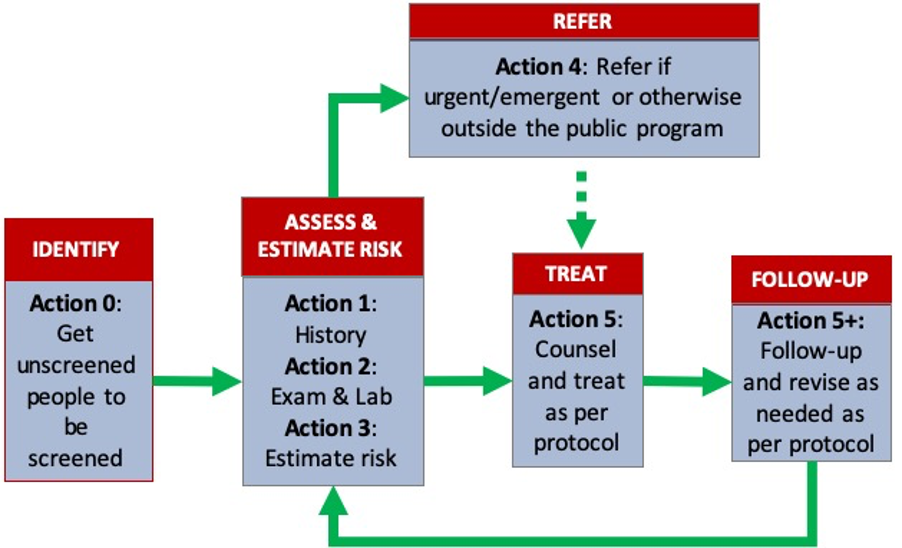


**Figure 2: WHO PEN core tasks**

**Table 2: List of tools**

| **Action number** | **Tool number** | **Tool name** | **Intended user** |
| --- | --- | --- | --- |
| **Action 0: Get unscreened people to be screened** | 0.1 | Patient ID card | Clinic staff |
|  | 0.2 | Risk screening form for Hypertension | Nurse, clinic staff |
| **Action 1: History-taking** | 1.1 | Treatment algorithm | Nurse, physician |
|  | 1.2 | Treatment card | Nurse, physician |
| **Action 2: Exam & lab** | 2 | Laboratory flowsheet  Diagnostic and treatment record sheet | Nurse, physician |
| **Action 3: Estimate risk** | 3 | Risk-based charts for Hypertension | Nurse, physician |
| **Action 4: Refer** | 4 | Referral form (referral institution) | Nurse, physician |
| **Action 5: Counsel and treat as per protocol** | 5.1 | Patient education material | Nurse, physician |
|  | 5.2 | Prescription form (general purpose) | Nurse, physician |
| **Action 6: Follow-up and revise as needed as per protocol** | 6.1 | Patient missed visit form | Nurse, clinic staff |
|  | 6.2 | Patient exit form | Clinic staff |

0.1. Patient ID card

0.2. Risk screening form

6.1 Patient missed visit form

6.2 Patient exit form

- 1. Treatment card

1.1 Treatment algorithm

1.2 Treatment card

3. Risk-based charts

4. Referral form (referral institution)

]

5.2. Prescription form

]

2. Laboratory flowsheet

4. Referral form (referral institution)

]

1.1 Treatment algorithm

- 1. Patient education material

]Bvc


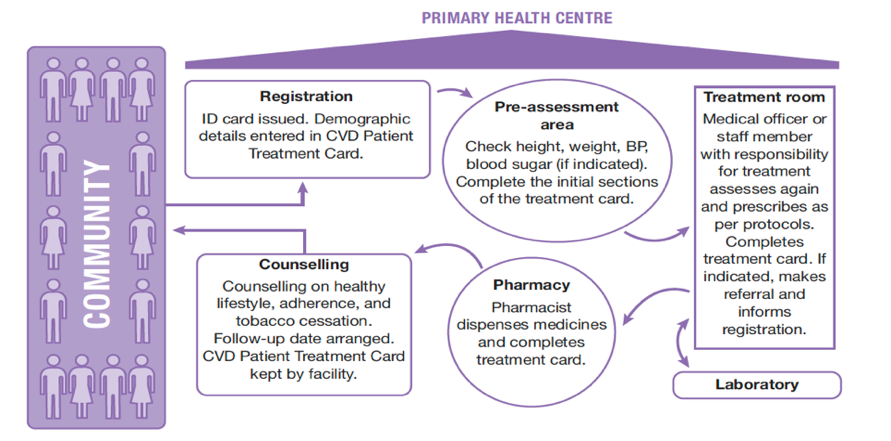


**Administration^1^**

Plan workflow; monitor and ensure adequate and timely supply of resources; monitor and report of outcomes

**REFFERAL HOSPITAL**

**Figure 3: Patient flow pathway^[[3]](#footnote-4)^**

*Adapted and translated into Khmer from the HEARTS Technical package for cardiovascular disease management in primary health care: systems for monitoring. Geneva: World Health Organization; 2018 (WHO/NMH/NVI/18.5). Licence: CC BY-NC-SA 3.0 IGO. WHO is not responsible for the content or accuracy of this translation. In the event of any inconsistency between the English and the Khmer translation, the original English version shall be the binding and authentic version.*

# 0.1. Patient ID card

The *patient ID card* is a card issued by clinic staff for new visitors to the HC and held by the patient. The ID card will have a unique HC ID issued to each patient by the HC, along with other personal and contact information. This card is to only to be issued once, at the time of initial registration at the HC. A duplicate copy of the ID card is kept by the clinic and the copy placed in a clinic registration file.

The patient shall bring this ID card to every clinic visit which clinic staff will then use to retrieve the patient’s treatment card kept at the HC using the unique HC ID. The patient ID card would, ideally, be linked to a national ID such as their Patient Management and Registration System (PMRS) or patient ID if available, along with information such as the health insurance / financing scheme they belong to or any means-tested benefits the patient may be eligible for at the time of payment. Alternatively, a national ID could be used to retrieve the same information if available.

The duplicate copy kept by the clinic is used to maintain a record of patients who have visited the clinic. HC staff can also use this card when contacting patients for follow-up visits.

| PATIENT ID CARD | |
| --- | --- |
| Date: | **Health Facility Code:** |
| Name of patient: | **Patient ID:** |
| Date of birth: | **Gender:** M F |
| Marital status: S M D W | **Health insurance:** |
| Street address: | **Village:** |
| Commune: | **District:** |
| Operational District: | **Province:** |
| Telephone number: | **Occupation**:  Farmer small business owner  office employee civil servant policeman soldier  construction worker housekeeper other unemployed |
| Remarks: | |

*Adapted from: Ministry of Health (2019). National Standard Operating Procedure for Diabetes and Hypertension Management in Primary Care. Department of Preventive Medicine. Phnom Penh, Ministry of Health*

# 0.2. Risk screening form

The *risk* *screening form* is to be filled in by clinic staff for all adult patients at their first visit and, subsequently, every five years.

The information collected in this form identifies whether a patient is a potential user of services aimed at treating DM and/or high BP as indicated in the *treatment algorithm (Tool 1.1)*. If they are, the attending nurse or physician shall proceed with filling in the *treatment card (Tool 1.2)* under the column “intake visit” either on the day itself or at a follow-up confirmatory visit.

The form is placed in a designated location in the patient HC file.

Note 1: for HCs with a collaboration with CHWs such as a PE or VHSG, this form can be used as a community screening form. Here the form would be filled by the CHW, with a copy of this form retained by the CHW and a duplicate copy shared with the HC. The HC and CHW would then coordinate to arrange follow-up visits at the HC or community level, as advised.

Note 2: The tests included in the risk screening form are those that can be conducted at point-of-care.

| RISK SCREENING FORM | |
| --- | --- |
| Date: | **Health Facility Code:** |
| Name of patient: | **Patient ID:** |
| Date of birth: | **Gender:** M F |
| Known hypertension: Y N | **Currently on antihypertensive medication:** Y N |
| Known diabetes: Y N | **Currently on diabetes medication:** Y N |
| Prior heart attack, heart disease or stroke: Y N | **Chronic kidney disease:** Y N |
| History heart attack, heart disease or stroke in first degree relatives: Y N | **History of diabetes or chronic kidney disease in first degree relatives:** Y N |
| Current smoker (used tobacco in the last 12 months): Y N | **Alcohol consumption:** N occasional daily harmful use |
| BP (systolic): | **BP (diastolic):** |
| Height (in meters): | **Weight (in kg):** |
| BMI: | **10-year CVD risk (refer to risk chart):** |
| Fasting blood glucose (mg/dl): | **Random blood glucose (mg/dl):** |
| Cholesterol: |  |
| Urine albumin: | **Urine ketones:** |
| Refer to referral hospital: Y N | **Date of HC evaluation intake visit:** |
| Name & signature: | |

*Adapted from: Ministry of Health (2019). National Standard Operating Procedure for Diabetes and Hypertension Management in Primary Care. Department of Preventive Medicine. Phnom Penh, Ministry of Health and WHO package of essential noncommunicable (PEN) disease interventions for primary health care. Geneva: World Health Organization; 2020. Licence: CC BY-NC-SA 3.0 IGO.*

# 1.1. Treatment algorithm (MOH Cambodia)

The *treatment algorithm* guides the attending nurse or physician to a treatment plan tailored to an individual based on their personal and familial history of CVD and assesses their lifestyle risk factors for the same. The healthcare provider will use the algorithm to determine whether the patient should be managed locally or referred to a higher-level facility. If managed locally, the algorithm guides the nurse or physician in managing the patient at the clinic using the information recorded in the *treatment card (Tool 1.2)*. The algorithm is designed based on the MOH’s National Standard Operating Procedure for Diabetes and Hypertension Management in Primary Care in Cambodia.

The treatment algorithm can be taped to a desk or a larger version of the algorithm and taped on a consultation wall.

1.2 Treatment card (facility)

The *treatment card* is filled in by the healthcare provider to whom the patient has been referred for evaluation.

The card documents essential information for monitoring treatment of CVD risk and tracking changes in health and treatment. To fulfill this function, the form is the place to record key data the provider needs to assess CVD risk using the *risk-based charts (Tool 3)* and follow the *treatment algorithm (Tool 1.1)*.

The first column of the CVD treatment card is the “intake visit”. Note that if the intake visit is the same as the screening visit, many of the entries will be the same as for the *risk screening form (Tool 0.2)*, and information can be copied directly. The intake visit may take place after the screening visit, as when an evaluation is scheduled for a separate day when a designated provider is available, or when the screening is done outside the HC in the community by a CHW. When the intake visit is on a later date than the screening visit, the provider should assess and record intake information based on their own observations.

A follow-up date for the next visit should be agreed upon and recorded in the card; this record can be used to schedule reminders for the next visit (either by phone or in person). Upon completion of the treatment card, the card shall be signed and dated by the attending nurse or physician at the HC.

The treatment card is to be placed in the “care encounter” section in the patient chart. Additional follow up visits are recorded on new pages.

At the time the first treatment card is used, the healthcare provider will also place an easily-visible coloured sticker on the outside of the patient chart to facilitate monitoring of clinic performance.

| TREATMENT CARD | | | |
| --- | --- | --- | --- |
| Health Facility Code: | | **Intake visit (visit 1)** | **Confirmation visit (visit 2)** |
| Patient ID: | | **Date:** | **Date:** |
| 1. Ask | **Previous diagnoses:** |  |  |
|  | Acute myocardial infarction | Y N | Y N |
|  | Angina /ischemic heart disease | Y N | Y N |
|  | Stroke | Y N | Y N |
|  | Transient ischemic attack | Y N | Y N |
|  | Hypertension (BP > 140/90) | Y N | Y N |
|  | Diabetes mellitus | Y N | Y N |
|  | Chronic kidney disease | Y N | Y N |
|  | **Current medications (name & daily dose):** |  |  |
|  | Medication 1 |  |  |
|  | Medication 2 |  |  |
|  | Medication 3 |  |  |
|  | Medication 4 |  |  |
|  | **Current smoker** | Y N | Y N |
| 2. Assess: | Weight |  |  |
|  | Height |  |  |
|  | BMI |  |  |
|  | BP (systolic) reading 1 |  |  |
|  | BP (diastolic) reading 1 |  |  |
|  | BP (systolic) reading 2 |  |  |
|  | BP (diastolic) reading 2 |  |  |
|  | Fasting blood glucose |  |  |
|  | Random blood glucose |  |  |
|  | HbA1c |  |  |
|  | Total cholesterol |  |  |
|  | Urine albumin |  |  |
|  | Urine ketones |  |  |
| 3. Estimate risk: | 10-year CVD risk |  |  |
| 4. Refer: | Refer to referral hospital | Y N | Y N |
| 5. Treat | **Counselling** |  |  |
|  | Counselling tobacco cessation | Y N | Y N |
|  | Counseling diet/physical activity | Y N | Y N |
|  | **Prescribed medications (name & daily dose):** |  |  |
|  | Medication 1 |  |  |
|  | Medication 2 |  |  |
|  | Medication 3 |  |  |
|  | Medication 4 |  |  |
| Date of next visit: | |  |  |
| Name & signature: | |  |  |

*Adapted and translated into Khmer by the KHANA Centre for Population Health Research from the WHO package of essential noncommunicable (PEN) disease interventions for primary health care. Geneva: World Health Organization; 2020. Licence: CC BY-NC-SA 3.0 IGO.WHO is not responsible for the content or accuracy of this translation. In the event of any inconsistency between the English and the Khmer translation, the original English version shall be the binding and authentic version.*

| TREATMENT CARD (CONTINUED) | | | |
| --- | --- | --- | --- |
| Health Facility Code: | | **Follow-up visit** | **Follow-up visit** |
| Patient ID: | | **Date:** | **Date:** |
| 1. Ask | **Previous diagnoses:** |  |  |
|  | Acute myocardial infarction | Y N | Y N |
|  | Angina /ischemic heart disease | Y N | Y N |
|  | Stroke | Y N | Y N |
|  | Transient ischemic attack | Y N | Y N |
|  | Hypertension (BP > 140/90) | Y N | Y N |
|  | Diabetes mellitus | Y N | Y N |
|  | Chronic kidney disease | Y N | Y N |
|  | **Current medications (name & daily dose):** |  |  |
|  | Medication 1 |  |  |
|  | Medication 2 |  |  |
|  | Medication 3 |  |  |
|  | Medication 4 |  |  |
|  | **Current smoker** | Y N | Y N |
| 2. Assess: | Weight |  |  |
|  | Height |  |  |
|  | BMI |  |  |
|  | BP (systolic) reading 1 |  |  |
|  | BP (diastolic) reading 1 |  |  |
|  | BP (systolic) reading 2 |  |  |
|  | BP (diastolic) reading 2 |  |  |
|  | Fasting blood glucose |  |  |
|  | Random blood glucose |  |  |
|  | HbA1c |  |  |
|  | Total cholesterol |  |  |
|  | Urine albumin |  |  |
|  | Urine ketones |  |  |
| 3. Estimate risk: | 10-year CVD risk |  |  |
| 4. Refer: | Refer to referral hospital | Y N | Y N |
| 5. Treat | **Counselling** |  |  |
|  | Counselling tobacco cessation | Y N | Y N |
|  | Counseling diet/physical activity | Y N | Y N |
|  | **Prescribed medications (name & daily dose):** |  |  |
|  | Medication 1 |  |  |
|  | Medication 2 |  |  |
|  | Medication 3 |  |  |
|  | Medication 4 |  |  |
| Date of next visit: | |  |  |
| Name & signature: | |  |  |

*Adapted and translated into Khmer by the KHANA Centre for Population Health Research from the WHO package of essential noncommunicable (PEN) disease interventions for primary health care. Geneva: World Health Organization; 2020. Licence: CC BY-NC-SA 3.0 IGO.WHO is not responsible for the content or accuracy of this translation. In the event of any inconsistency between the English and the Khmer translation, the original English version shall be the binding and authentic version.*

# 2. Laboratory flowsheet

The *laboratory flowsheet* is to be filled in by the healthcare provider based on point-of-care testing or outside laboratory results. The data elements are focused on the information required to follow the *treatment algorithm (Tool 1.1)* for managing elevated BP. The provider transcribing the data to the sheet must sign and date each column entry.

The form is to be kept in a “laboratory” section of the patient chart with all other charts filed in reverse chronological order.

Note 1: this flowsheet only focuses on HT management; additional entries can be included if the HC becomes directly involved in managing a DM plan.

Note 2: this flowsheet is to be used for HCs that have access to outside or more advanced lab testing.

| LABORATORY FLOWSHEET | | | | | | |
| --- | --- | --- | --- | --- | --- | --- |
| Health Facility Code: | | | | **Sampling date:** | | |
| Patient ID: | | | | **Result date:** | | |
| Name of patient: | | | | **Request no:** | | |
|  |  |  |  | **Result no:** | | |
| Date of birth: | | | | **Gender:** M F | | |
| No | **Test description** | **Date 1** | **Date 2** | **Date 3** | **Unit** | **Normal range** |
| 1 – BLOOD TEST RESULT | | | | | | |
| 1 | Fasting blood glucose |  |  |  | mg/dl |  |
| 2 | Random blood glucose |  |  |  | mg/dl |  |
| 2 | HbA1c |  |  |  | % |  |
| 3 | Triglyceride |  |  |  | mg/dl |  |
| 4 | Total cholesterol |  |  |  | mg/dl |  |
| 5 | HDL cholesterol |  |  |  | mg/dl |  |
| 6 | Estimated LDL cholesterol |  |  |  | mg/dl |  |
| 7 | Creatinine |  |  |  | mg/dl |  |
| 8 | eGFR |  |  |  | mL/min |  |
| II – URINE TEST RESULT | | | | | | |
| 1 | Proteinuria |  |  |  |  |  |
| 2 | Albuminuria |  |  |  | mg/l |  |
| 3 | Urine creatinine |  |  |  | mg/dl |  |
| 4 | Albumin-to-creatinine ratio |  |  |  |  |  |
| 5 | Urine ketones |  |  |  |  |  |
| Name and signature | |  |  |  |  | |

*Adapted from MoPoTsyo Patient Information Centre laboratory form, Cambodia*

# 3. Risk-based charts

The *risk-based charts* are CVD risk non-laboratory-based charts published in the WHO HEARTS package (World Health Organization 2018) and translated into Khmer for local use at the HC. The attending nurse or physician estimates the risk of a patient developing CVD considering a patient’s age, sex, smoking status, systolic BP and body mass index (BMI). The estimated risk then feeds into the *treatment algorithm (Tool 1.1)* which will inform the healthcare provider of the appropriate course of action to take.

The charts can be taped to a desk or a larger version of the charts printed and taped on a consultation wall.

Note 1: if laboratory results with lipids are available, non-laboratory-based risk charts can be replaced with laboratory-based charts in the future.


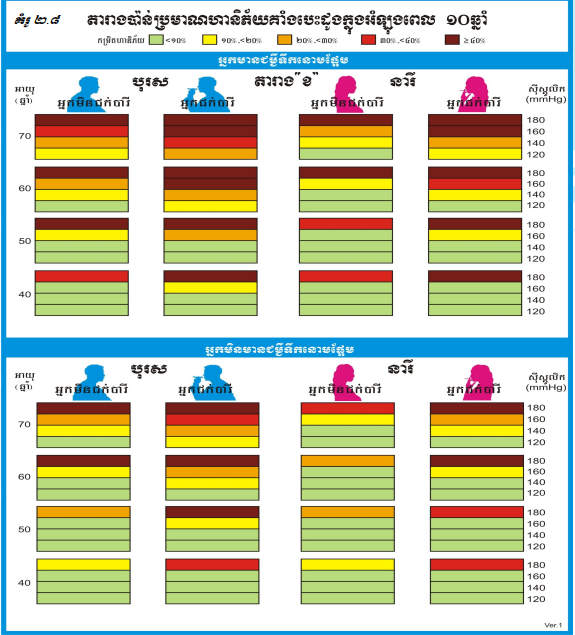


*Reproduced from: Ministry of Health (2019). National Standard Operating Procedure for Diabetes and Hypertension Management in Primary Care. Department of Preventive Medicine. Phnom Penh, Ministry of Health*

# 4. Referral form (referral institution)

The referral institution (RI) is a designated centre for receiving HT or DM patients that are deemed by the algorithm to require more advanced evaluations, either a clinic or a hospital. Alternatively, the HC may have a special clinic located at the HC that will take NCD patients on designated days.

The *referral form* is filled in by the attending nurse or physician to refer patients to a higher-level facility as indicated in the *treatment algorithm (Tool 1.1)*. The healthcare provider fills out the first section of the form “referral form to referral institution” to record present diagnoses, signs/symptoms, self-reported complications, current medications (if any) and measurements taken at the time of visit. A copy of the form shall be retained by the HC and a duplicate copy shared with the patient who can use this to schedule a visit to the RI. Alternatively, the HC can scan and electronically transfer (using an approved standard social medium) the completed form to the RI if such facilities are available at both sites of care.

At the RI, the attending physician shall fill in the second section of the form “feedback from referral institution” to confirm diagnosis, prescribe medications (if any) and note follow-up requirements at either the RI or HC. A copy of the form shall be retained by the RI and a duplicate copy shared with the patient who can then share the copy at their next scheduled visit to the HC. Alternatively, the RI can scan and electronically transfer the completed form to the HC if such facilities are available at both sites of care.

The intended use of this form is to facilitate appointments made at the HC for a visit to a RI. Ideally, the RI would receive a copy of the referral form in advance of the patient’s visit, and would, likewise, send a copy of the feedback form back to the HC in advance of the patient’s follow-up visit at the HC. If this is not possible, a modification will need to be made to simplify the process.

This form will be placed in the “care encounters” section of the patient chart.

| REFERRAL FORM TO REFERRAL INSTITUTION | |
| --- | --- |
| Date: | **Health Facility Code:** |
| Name of patient: | **Patient ID:** |
| Date of birth: | **Gender:** M F |
| Current diagnoses / signs & symptoms / measurements / complications: | |
| Medications: | |
| Reason(s) for referral: | |
| Name of referral institution: | |
| Name & signature: | |
| FEEDBACK FROM REFERRAL INSTITUTION | |
| Date: | **Name of referral institution:** |
| Diagnosis: | |
| Medications: | |
| Follow-up requirements: | |
| Remarks: | |
| Name & signature: | |

*Adapted and translated into Khmer by the KHANA Centre for Population Health Research from the World Health Organization (2013). Implementation tools: Package of Essential Noncommunicable (PEN) disease interventions for primary health care in low-resource settings. Luxembourg, World Health Organization. WHO is not responsible for the content or accuracy of this translation. In the event of any inconsistency between the English and the Khmer translation, the original English version shall be the binding and authentic version.*

# 5. Counsel and Treat

# 5.1 Patient education material

The following patient education material is filled in by the attending nurse or physician to counsel all patients on risk factors for CVD and how to recognise symptoms for the same. The material also includes information on how lifestyle changes in diet, exercise and smoking can be used to lower one’s risk of developing CVD disease. A user manual can be developed to instruct health providers on how to use such materials in counselling patients.

NCD awareness and prevention posters can be taped to a consultation wall or showcased at visible places at the health centre. NCD leaflets can also be shared with patients to take home with them if sufficient resources are available.

1. **Sample leaflet for high blood pressure awareness raising**


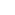


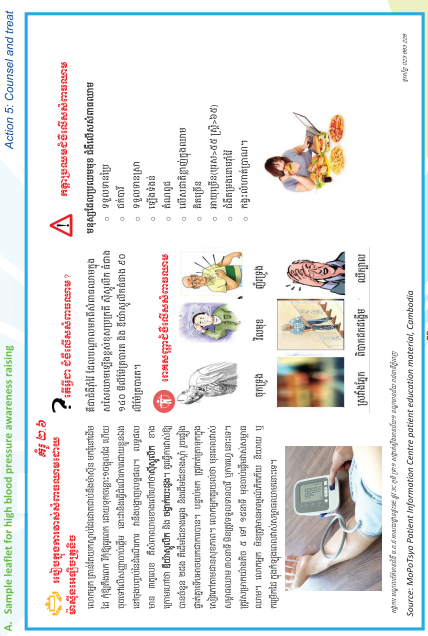


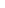


*Source*: *MoPoTsyo Patient Information Centre patient education material, Cambodia*

1. **Sample leaflet for diabetes awareness raising**


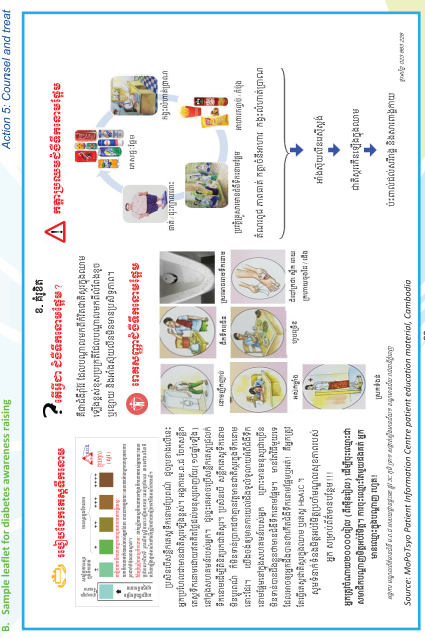

*Source*: *MoPoTsyo Patient Information Centre patient education material, Cambodia*

1. **Sample NCD primary prevention poster**

*
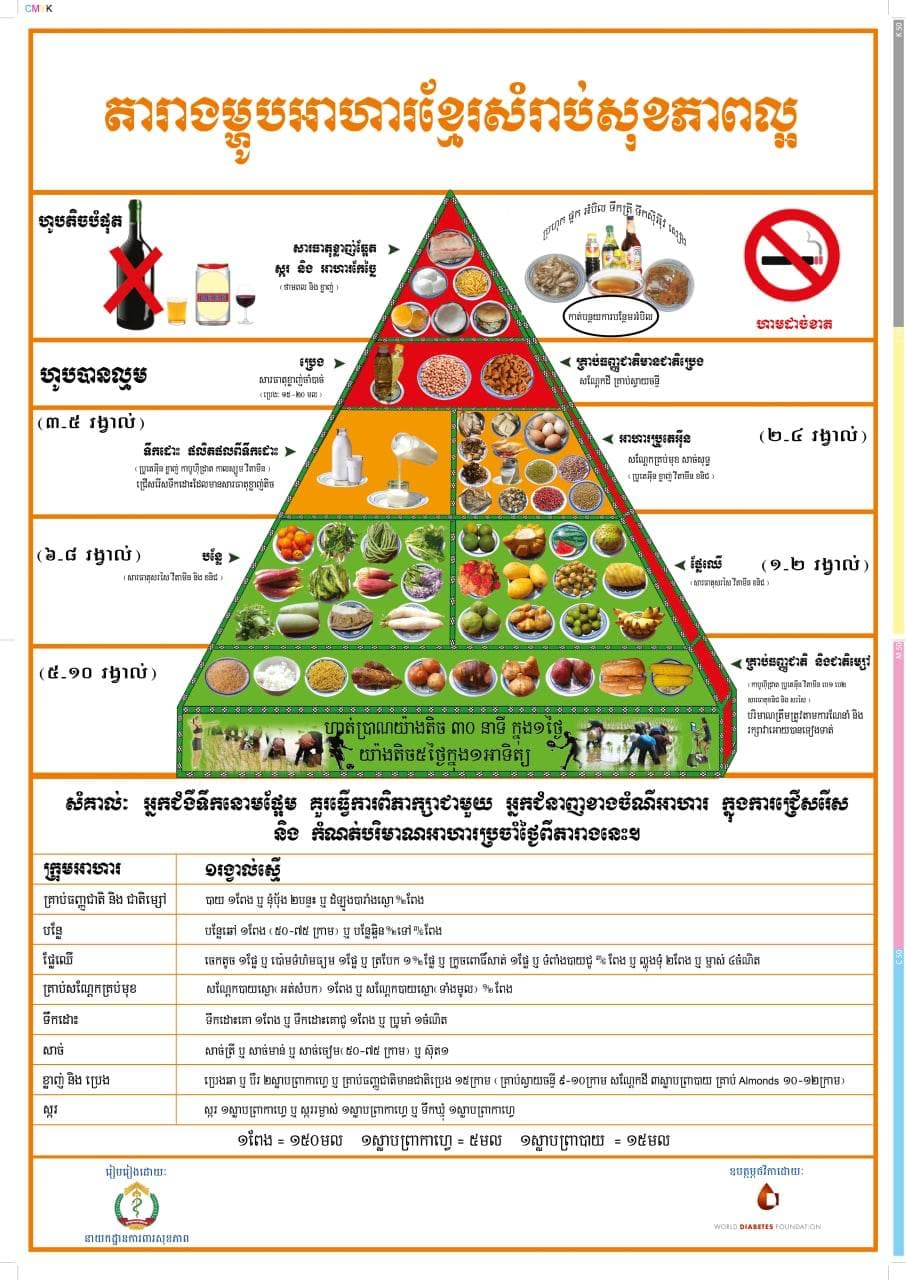
*

*Source: Patient education material of the Preventive Medicine Department, Ministry of Health, Cambodia*

1. **Sample high Blood Pressure pyramid poster**


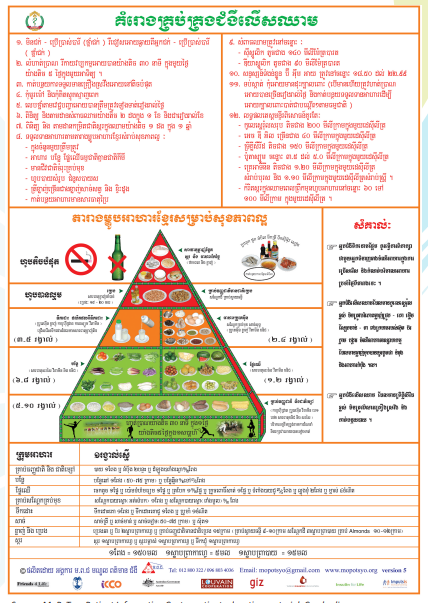


*Source*: *MoPoTsyo Patient Information Centre patient education material, Cambodia*

1. **Sample diabetes pyramid poster**

*Source*: *MoPoTsyo Patient Information Centre patient education material, Cambodia*

1. **Sample potassium poster**


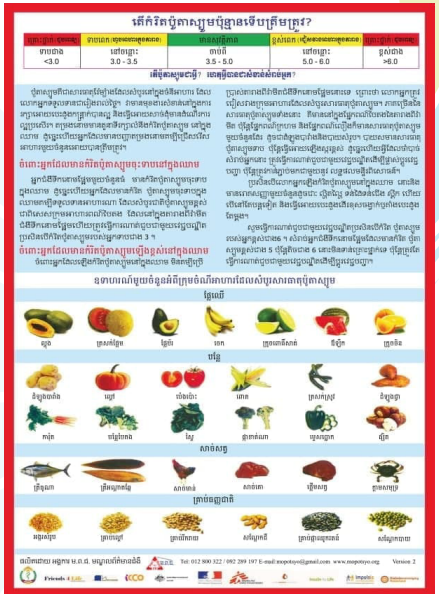


*Source*: *MoPoTsyo Patient Information Centre patient education material, Cambodia*

# 5.2. Prescription form (general purpose)

The *prescription form* is filled in by the attending nurse or physician to prescribe medications to patients visiting the HC. The form can be customised based on which medications are recommended in the *treatment algorithm (Tool 1.1)* and/or available at the HC. The form can also be filled in by the healthcare provider to purchase medications outside the HC such as a private pharmacy, for example, if unavailable in-house. The prescription form will be handed to and retained by the pharmacist and a receipt issued to patient on receipt of payment.

Note 1: The tool assumes that the healthcare provider at the HC is authorised to write prescriptions under the guidance of a physician at a RI or at the HC. If the healthcare provider is unauthorised to write prescriptions for any of the drugs listed in the prescription form, please delete accordingly. Further, if any drugs not currently listed in the prescription form can be prescribed, these medications should be added to the list as well.

Note 2: Drugs included in the form would have to be consistent with what is locally available either through the Central Medical Store (CMS) or at a private pharmacy.

| PRESCRIPTION FORM | | | | | |
| --- | --- | --- | --- | --- | --- |
| Date: | | **Health Facility Code:** | | | |
| Name of patient: | | **Patient ID:** | | | |
| No | **Drug** | | **Morning** | **Afternoon** | **Night** |
| 1 | Amitriptyline (12.5 mg / 25 mg) | |  |  |  |
| 2 | Amlodipine (5 mg / 10 mg) | |  |  |  |
| 3 | Aspirin 300 mg (150 mg / 300 mg) | |  |  |  |
| 4 | Atenolol (25 mg / 50 mg) | |  |  |  |
| 5 | Enalapril (10 mg / 20 mg) | |  |  |  |
| 6 | Furosemide (20 mg / 40 mg) | |  |  |  |
| 7 | **Hydrochlorothiazide (12.5 mg /25 mg)** | |  |  |  |
| 8 | Losartan Potassium (50 mg / 100mg) | |  |  |  |
| 9 | Glibenclamide (2.5 mg / 5 mg) | |  |  |  |
| 10 | Insulin Actrapid 1ml=100units | |  |  |  |
| 11 | Insulin Mixtard 30/70 1ml=100units | |  |  |  |
| 12 | Insulin NPH 1ml=100units | |  |  |  |
| 13 | Metformin (500 mg / 2000 mg) | |  |  |  |
| 14 | Simvastatin (10 mg / 20 mg) | |  |  |  |
| 15 | Multivitamin | |  |  |  |
| 16 | Thiamine (VitB1) (25 mg / 50mg | |  |  |  |
| 17 | Other medication 1 (please specify): | |  |  |  |
| 18 | Other medication 2 (please specify): | |  |  |  |
| Advice | | | | | |
| Name and signature | | | | | |

*Adapted from: MoPoTsyo Patient Information Centre prescription form, Cambodia*

# 6. Forms for missed visits

# 6.1. Patient missed visit form

The *patient missed visit form* is filled in by clinic staff to record missed visits at the health centre. It can be also be used to keep track of rescheduled visits. The form assumes that contact can be made via a phone call; this can be modified to accommodate other means of contact including text messages or house visits, for example.

This form should be kept in the “care encounters” section of the patient chart.

| PATIENT MISSED VISIT FORM | |
| --- | --- |
| Date: | **Health Facility Code:** |
| Name of patient: | **Patient ID:** |
| Telephone number: | **Name of clinic staff:** |
| Outcome of phone call:   1. Visit rescheduled to: ___ / ___ / ___ 2. Patient not reached (no answer) 3. Phone not reached 4. Patient exited (died or LTFU^[[4]](#footnote-5)^– complete exit form) 5. Other:_____________________ | **Reason patient missed visit:**   1. Unable to afford transportation 2. Illness (too unwell to transfer to facility) 3. Did not remember visit date 4. Did not think it was necessary to come 5. Declined care 6. Seen at different health facility 7. Transferred (complete exit form) 8. Other:_____________________ |
| Name & signature: | |

*Adapted from: Partners in Health PEN-Plus Toolkit call back form*

# 6.2. Patient exit form

The *patient exit form* is filled in by clinic staff to de-register patients at the HC for various reasons, including death, LTFU or patients declining care. If LTFU, the HC shall determine what would be an appropriate definition to assign to the same.

This form would be attached to the *patient ID card (Tool 0.1)* in the clinic registration file.

| PATIENT EXIT FORM | |
| --- | --- |
| Date of exit: | **Health Facility Code:** |
| Name of patient: | **Patient ID:** |
| Name of health worker: |  |
| Reason for exit:   1. Death 2. Lost to follow-up^[[5]](#footnote-6)^ 3. Declined care 4. Other: _____________________ | **Location of death (if applicable):**   1. Home 2. While admitted to hospital (health facility name): _________________ 3. Other death location___________ |
| Name & signature: | |

*Adapted from: Partners in Health PEN-Plus Toolkit patient exit form*

# Appendix

The following set of the tools in the Appendix are tools that can be used by HCs as appropriate based on care guidelines and relationships with other healthcare providers specific to each HC. Some of these tools taken from HEARTS Technical Package and translated into Khmer for local adaptation and use in Cambodia.

| **Tool number** | **Tool name** | **Intended user** |
| --- | --- | --- |
| A1 | Referral form (community health worker) | Nurse, physician |
| A2 | Employee sign-in sheet | Clinic staff |
| A3 | Stock card | Clinic staff |
| A4 | Technology needs assessment | Clinic staff |
| A5 | Medicines needs assessment | Clinic staff |
| A6 | Purchase order | Clinic staff |
| A7 | Budget sheet | Clinic staff |
| A8 | Cashflow projection sheet | Clinic staff |
| A9 | Facility register for monitoring outcomes | Clinic staff |
| A10 | Health facility report | Clinic staff |
| A11 | Patient report card | District/provincial-level manager |
| A12 | Treatment supervision/audit form | District/provincial-level manager |
| A13 | Summary of supervision visits | District/provincial-level manager |
| A14 | Pre-visit planning: Workflow mapping worksheet | Clinic staff, district/provincial-level manager |
| A15 | Pre-visit planning: Assessment of current practice | Clinic staff, district/provincial-level manager |
| A16 | Patient visit: Workflow mapping worksheet | Clinic staff, district/provincial-level manager |
| A17 | Patient visit: Assessment of current practice | Clinic staff, district/provincial-level manager |
| A18 | Post-visit follow-up: Workflow mapping worksheet | Clinic staff, district/provincial-level manager |
| A19 | Post-visit follow-up: Assessment of current practice | Clinic staff, district/provincial-level manager |

## A1. Referral form (community health worker)

The *referral form (CHW)* is filled in the attending nurse or physician at the HC to refer patients to a CHW (such as a PE or VHSG), where advisable, for monitoring health behaviours and outcomes. The healthcare provider shall complete the first part of the form “referral form to community health worker” including measurements, diagnoses and medications prescribed.

A copy of the form shall be retained by the HC and a duplicate copy shared with the CHW who can use this to contact and schedule a visit with the patient in their community.

During the visit, the CHW will fill in the second half of the form “feedback from community health worker” to record any measurements taken and counselling advice provided as per the *treatment algorithm (Tool 1.1)* and *patient education material (Tool 5.1).* A copy of the form shall be retained by the CHW and a duplicate copy shared with the patient who can then share the copy at their next scheduled visit to the HC. Alternatively, the CHW can scan, fax or email a copy of the completed form to the HC if such facilities are available.

| REFERRAL FORM TO COMMUNITY HEALTH WORKER | |
| --- | --- |
| Date: | **Health Facility Code:** |
| Name of patient: | **Patient ID:** |
| Date of birth: | **Gender:** M F |
| Marital status: S M D W | **Health insurance:** |
| Street address: | **Village:** |
| Commune: | **District:** |
| Operational District: | **Province:** |
| Telephone number: | **Occupation**:  Farmer small business owner  office employee civil servant policeman soldier  construction worker housekeeper other unemployed |
| Measurements / diagnosis: | |
| Complications: | |
| Medications: | |
| Name of referral institution: | |
| Remarks: | |
| Name & signature: | |
| FEEDBACK FROM COMMUNITY HEALTH WORKER | |
| Date: | |
| Measurements taken: | |
| Lifestyle advice given: | |
| Follow-up remarks: | |
| Name & signature: | |

## A2. Employee sign-in sheet

The daily *employee sign-in sheet* is used to track HC staff presenteeism and absenteeism rates and take corrective action if and when needed. The employee shall clock their time in and time out of the clinic, which will be reviewed and signed by a supervisor to confirm the same.

| EMPLOYEE SIGN-IN SHEET | | | | | |
| --- | --- | --- | --- | --- | --- |
| Date | **Name** | **Time In** | **Time Out** | **Initials** | **Supervisor Initial** |
|  |  |  |  |  |  |
|  |  |  |  |  |  |
|  |  |  |  |  |  |
|  |  |  |  |  |  |
|  |  |  |  |  |  |
|  |  |  |  |  |  |
|  |  |  |  |  |  |
|  |  |  |  |  |  |
|  |  |  |  |  |  |
|  |  |  |  |  |  |
|  |  |  |  |  |  |
|  |  |  |  |  |  |
|  |  |  |  |  |  |
|  |  |  |  |  |  |

*Adapted from: eForms. (2020). "Employee Sign-in Sheet Template." Retrieved 19 October 2020, n.d., from* [*https://eforms.com/sign-in-sheet/employee/.*](https://eforms.com/sign-in-sheet/employee/.)

## A3. Stock card

The *stock card* template is filled in by clinic staff to record incoming and outgoing stock of medicines and diagnostic equipment and monitor stock levels on a daily, weekly or monthly basis, as appropriate. Tracking supplies will ensure staff know when new purchases needed to be made based on the minimum and maximum stock levels required. The minimum stock level can be determined using the *technology needs assessment* *(Tool A4)* and *medicines needs assessment (Tool A5) forms* below. New purchase orders can be made using the *purchase order/voucher* *(Tool A6)* below when stocks hit the minimum stock level in the stock card (or, equivalently, the reorder level stock (D) in the *technology needs assessment* and *medicines needs assessment* forms).

| STOCK CARD | | | | | | | | | |
| --- | --- | --- | --- | --- | --- | --- | --- | --- | --- |
| **Item description (name, formulation, strength):** Glibenclamide, 5 mg | | | | | |  | **Code number:** | | |
|  |  |  |  |  |  |  |  |  |  |
| **Unit + size:** tabs | | | | | |  | **Special storage conditions:** | | |
|  |  |  |  |  |  |  |  |  |  |
| **Maximum stock level:** | | | | | | **Minimum stock level:** | | | |
| **Date** | **Received from** | **Voucher number** | **Quantity received** | **Issued to** | **Quantity issued** | **Losses/adjustments** | **Balance in stock** | **Remarks** | **Initials** |
|  |  |  |  |  |  |  |  |  |  |
| 1/1/20 | CMS | 100ABC | 100,000 |  |  |  |  |  |  |
| 7/1/20 |  |  |  | Pharmacy | 1000 |  | 99,000 | 1/1/20-7/1/20 | NS |
| 14/1/20 |  |  |  | Pharmacy | 800 |  | 98,200 | 8/1/20-14/1/20 | NS |
|  |  |  |  |  |  |  |  |  |  |

*Adapted and translated into Khmer by the KHANA Centre for Population Health Research from the Operations Manual for Delivery of HIV Prevention, Care and Treatment*

*at Primary Health Centres in High Prevalence, Resource-Constrained Settings. Geneva, WHO and Infectious Diseases Research Collaboration and L. S. o. H. T. M. ACT Consortium (2011). The ACT PRIME Study Learner Manual: Health Centre Management - Drug Supply Management. London, The ACT Consortium. World Health Organization, 2008. WHO is not responsible for the content or accuracy of this translation. In the event of any inconsistency between the English and the Khmer translation, the original English version shall be the binding and authentic version.*

## A4. Technology needs assessment

The *technology needs assessment* form is filled in by clinic staff to estimate stock levels (D) of equipment at which reorders need to be made to ensure adequate stocks are available given average consumption (A), safety stock levels (C) and average lead times (the time between placing an order and receiving it). Stock balances as monitored in the *Stock card* *(Tool A3)* can be used to record current stock levels (B) on a periodic basis.

| **TECHNOLOGY NEEDS ASSESSMENT** | | | | | | | | | | | | | |
| --- | --- | --- | --- | --- | --- | --- | --- | --- | --- | --- | --- | --- | --- |
| **No** | **Items** | **Unit** | **Form** | **Unit cost US$** | **Average consumption per month** | **Current stock** | **Safety stock** | **Stock issued during lead time** | **Reorder level** | **Order quantity for 6 months** | **Quantity to be reordered** | **Estimated cost in US$** |  |
|  |  |  |  |  | **A** | **B** | **2 x A = C** | **3 x A = D** | **E = C + D** | **F = 6 x A** | **G = (E + F) - B** | **G x (Unit Cost)** |  |
| **Essential technologies/lab investigations/tools** | | | | | | | | | | | | | |
| 1 | Stethoscope | 1 | pcs | 5 | 1 | 1 | 2 | 3 | 5 | 6 | 10 | 50 |  |
| 2 | Blood pressure measurement device | 1 | pcs | 5 | 1 | 1 | 2 | 3 | 5 | 6 | 10 | 50 |  |
| 3 | Measuring tape | 1 | pcs | 5 | 1 | 1 | 2 | 3 | 5 | 6 | 10 | 50 |  |
| 4 | Weighing machine (scale) | 1 | pcs | 5 | 1 | 1 | 2 | 3 | 5 | 6 | 10 | 50 |  |
| 5 | BMI chart | 1 | pcs | 5 | 1 | 1 | 2 | 3 | 5 | 6 | 10 | 50 |  |
| 6 | Glucometer | 1 | pcs | 5 | 1 | 1 | 2 | 3 | 5 | 6 | 10 | 50 |  |
| 7 | Blood glucose test strips | 100 | strips | 10 | 1 | 1 | 2 | 3 | 5 | 6 | 10 | 100 |  |
| 8 | Urine albumin test strips | 100 | strips | 10 | 1 | 1 | 2 | 3 | 5 | 6 | 10 | 100 |  |
| 9 | Urine ketones test strips | 100 | strips | 10 | 1 | 1 | 2 | 3 | 5 | 6 | 10 | 100 |  |
| Other |  |  |  |  |  |  |  |  |  |  |  |  |  |
| 10 | Blood cholesterol assay | 1 |  | 5 | 1 | 0 | 2 | 3 | 5 | 6 | 11 | 55 |  |
| 11 | Lipid profile | 1 |  | 5 | 1 | 0 | 2 | 3 | 5 | 6 | 11 | 55 |  |
| 12 | Serum creatinine assay | 1 |  | 5 | 1 | 0 | 2 | 3 | 5 | 6 | 11 | 55 |  |
| 13 | Serum potassium | 1 |  | 5 | 1 | 0 | 2 | 3 | 5 | 6 | 11 | 55 |  |
| 14 | Haemoglobin a1c | 1 |  | 5 | 1 | 0 | 2 | 3 | 5 | 6 | 11 | 55 |  |
| 15 | Urine microalbuminuria test strips | 1 | strips | 5 | 1 | 0 | 2 | 3 | 5 | 6 | 11 | 55 |  |
| **Total estimated order value** | |  |  |  |  |  |  |  |  |  |  | **930** |  |

*Adapted from: Suy Vannak (2010). Evaluation of the Revolving Drug Fund Project of Patient Information Centre. Master of Business Administration Research Report, Norton University and the HEARTS Technical package for cardiovascular disease management in primary health care: implementation guide. Geneva: World Health Organization; 2018 (WHO/NMH/NVI/18.14). Licence: CC BYNC- SA 3.0 IG*

## A5. Medicines needs assessment

The *medicines needs assessment* form is filled in by clinic staff to estimate the minimum stock levels (D) of medicines at which reorders need to be made to ensure adequate stocks are available given average consumption (A), safety stock levels (C) and average lead times (the time between placing an order and receiving it). Stock balances as monitored in the *Stock card* *(Tool A3)* can be used to record current stock levels (B) on a periodic basis.

| **MEDICINES NEEDS ASSESSMENT** | | | | | | | | | | | | | | |
| --- | --- | --- | --- | --- | --- | --- | --- | --- | --- | --- | --- | --- | --- | --- |
| **No** | **Items** | **Dosage** | **Unit** | **Form** | **Unit cost US$** | **Average consumption per month** | **Current stock** | **Safety stock** | **Stock issued during lead time** | **Reorder level** | **Order quantity for 6 months** | **Quantity to be reordered** | **Estimated cost in US$** |  |
|  |  |  |  |  |  | **A** | **B** | **2 x A = C** | **3 x A = D** | **E = C + D** | **F = 6 x A** | **G = (E + F) - B** | **G x (Unit Cost)** |  |
| 1 | Glibenclamide | 5mg | 1000 | tabs | 0.01 | 10,396 | 8,200 | 20,792 | 31,188 | 51,980 | 62,376 | 106,156 | 1,062 |  |
| 2 | Metformin | 500mg | 500 | tabs | 0.02 | 13,179 | 8,900 | 26,358 | 39,537 | 65,895 | 79,074 | 136,069 | 2,721 |  |
| 3 |  |  |  |  |  |  |  |  |  |  |  |  |  |  |
| 4 |  |  |  |  |  |  |  |  |  |  |  |  |  |  |
| 5 |  |  |  |  |  |  |  |  |  |  |  |  |  |  |
| **Total estimated order value** | | |  |  |  |  |  |  |  |  |  |  | **3,783** |  |

*Adapted from: Suy Vannak (2010). Evaluation of the Revolving Drug Fund Project of Patient Information Centre. Master of Business Administration Research Report, Norton University.*

## A6. Purchase order/voucher

The *purchase order / voucher* template is filled in by clinic staff to purchase new medicines and/or equipment from vendors when stocks hit the minimum stock level in the *stock card* *(Tool A3)* (or, equivalently, the reorder level stock (D) in the *technology needs assessment (Tool A4)* and *medicines needs assessment* *(Tool A5)* forms).

| PURCHASE ORDER/VOUCHER | |
| --- | --- |
| Purchase Order No.: | |
| Date: | |
| Goods: | **Amount:** |
|  |  |
|  |  |
|  |  |
| Authorized by (health centre) | |
| Created by (allotment number) | |

*Adapted and translated into Khmer by the KHANA Centre for Population Health Research from the Operations*

*Manual for Delivery of HIV Prevention, Care and Treatment at Primary Health Centres in High Prevalence,*

*Resource-Constrained Settings. Geneva, WHO and Infectious Diseases Research Collaboration and L. S. o. H. T.*

*M. ACT Consortium (2011). The ACT PRIME Study Learner Manual: Health Centre Management - Drug Supply*

*Management. London, The ACT Consortium. World Health Organization, 2008. WHO is not responsible for the content or accuracy of this translation. In the event of any inconsistency between the English and the Khmer translation, the original English version shall be the binding and authentic version.*

## A7. Budget sheet

The *budget sheet* is filled in by clinic staff to keep track of total projected and actual incomes and expenditures by the HC on a monthly, quarterly or annual basis as so desired. Line item categories would include personnel, equipment and medical supplies and can be modified based on the resource requirements of each HC.

| BUDGET SHEET | | | | | | | | | |
| --- | --- | --- | --- | --- | --- | --- | --- | --- | --- |
| Expenditures | | | | | | **Income** | | | |
| Category | **Resources needed** | **Costing Unit** | **Cost per unit** | **Number of units** | **Total costs** | **Donor A** | **Donor B** | **Ministry** | **Total income** |
| Personnel | Nurses | Monthly salary | 300 | 24 | 7200 |  |  |  |  |
|  | Driver | Monthly salary | 100 | 12 | 1200 |  |  |  |  |
|  | Subtotal |  |  |  | 8400 |  |  |  |  |
| Equipment | Xxxx | xxxx | xxxx | xxxx | xxxx |  |  |  |  |
|  | Xxxx | xxxx | xxxx | xxxx | xxxx |  |  |  |  |
|  | Subtotal |  |  |  | xxxx |  |  |  |  |
| Medical supplies | Xxxx | xxxx | xxxx | xxxx | xxxx |  |  |  |  |
|  | Xxxx | xxxx | xxxx | xxxx | xxxx |  |  |  |  |
|  | Subtotal |  |  |  | xxxx |  |  |  |  |
| GRAND TOTAL |  |  |  |  | **xxxx** | **xxxx** | **xxxx** | **xxxx** | **xxxx** |

*Adapted and translated into Khmer by the KHANA Centre for Population Health Research from the Operations Manual for Delivery of HIV Prevention, Care and Treatment at Primary Health*

*Centres in High Prevalence, Resource-Constrained Settings. Geneva, WHO and Infectious Diseases Research Collaboration and L. S. o. H. T. M. ACT Consortium (2011). The ACT PRIME Study*

*Learner Manual: Health Centre Management - Drug Supply Management. London, The ACT Consortium. World Health Organization, 2008. WHO is not responsible for the content or accuracy of this translation. In the event of any inconsistency between the English and the Khmer translation, the original English version shall be the binding and authentic version.*

## A8. Cashflow projection sheet

The *cashflow projection sheet* is filled in by clinic staff to project and monitor cashflows on a monthly, quarterly or annual basis as so desired taking into account projected income and expenditures.

| CASHFLOW PROJECTION SHEET | | | | | | | | | | | | | |
| --- | --- | --- | --- | --- | --- | --- | --- | --- | --- | --- | --- | --- | --- |
| Category | **Jan** | **Feb** | **Mar** | **Apr** | **May** | **Jun** | **Jul** | **Aug** | **Sep** | **Oct** | **Nov** | **Dec** |  |
| Funds at beginning of month |  |  |  |  |  |  |  |  |  |  |  |  |  |
|  |  |  |  |  |  |  |  |  |  |  |  |  |  |
| Projected income |  |  |  |  |  |  |  |  |  |  |  |  |  |
| Donor A |  |  |  |  |  |  |  |  |  |  |  |  |  |
| Donor B |  |  |  |  |  |  |  |  |  |  |  |  |  |
| Ministry |  |  |  |  |  |  |  |  |  |  |  |  |  |
| Total income |  |  |  |  |  |  |  |  |  |  |  |  |  |
|  |  |  |  |  |  |  |  |  |  |  |  |  |  |
| Projected Expenditures |  |  |  |  |  |  |  |  |  |  |  |  |  |
| Personnel |  |  |  |  |  |  |  |  |  |  |  |  |  |
| Equipment |  |  |  |  |  |  |  |  |  |  |  |  |  |
| Medical supplies |  |  |  |  |  |  |  |  |  |  |  |  |  |
| Total Expenditures |  |  |  |  |  |  |  |  |  |  |  |  |  |
|  |  |  |  |  |  |  |  |  |  |  |  |  |  |
| Funds available at end of month* |  |  |  |  |  |  |  |  |  |  |  |  |  |

*=funds at the beginning month + total income – total expenditure

*Adapted and translated into Khmer by the KHANA Centre for Population Health Research from the Operations Manual for Delivery of HIV Prevention, Care and Treatment*

*at Primary Health Centres in High Prevalence, Resource-Constrained Settings. Geneva, WHO and Infectious Diseases Research Collaboration and L. S. o. H. T.*

*M. ACT Consortium (2011). The ACT PRIME Study Learner Manual: Health Centre Management - Drug Supply Management. London, The ACT Consortium. World Health*

*Organization, 2008. WHO is not responsible for the content or accuracy of this translation. In the event of any inconsistency between the English and the Khmer translation, the original English version shall be the binding and authentic version.*

## A9. Facility register for monitoring outcomes

The *facility register for monitoring outcomes* is filled in by clinic staff to track management of patients with elevated BP based on the information in the *treatment card (Tool 1.2)*. This register will be filled in after every visit of a patient diagnosed with elevated BP. The information recorded will be summarised in a *health facility report (Tool A10)* submitted to district/provincial-level facilities at regular intervals to monitor progress and revisit current management practices as and when needed.

This could also be done retrospectively based on an audit of charts with coloured stickers at specified intervals.

| FACILITY REGISTER FOR MONITORING OUTCOMES | | | | | | | |
| --- | --- | --- | --- | --- | --- | --- | --- |
| Health Facility Code (or PRMS ID if available): | **Gender (M/F)** | **D.O.B (dd/mm/yy)** | **Date of registration** | **Visit date** | **BP measurement** | | **BP controlled at initial visit? Y/N** |
|  |  |  |  |  | **S** | **D** |  |
| 001 |  |  |  |  |  |  |  |
| 002 |  |  |  |  |  |  |  |
| 003 |  |  |  |  |  |  |  |
| 004 |  |  |  |  |  |  |  |
| 002 |  |  |  |  |  |  |  |
| 001 |  |  |  |  |  |  |  |

*Reproduced and translated into Khmer from the HEARTS Technical package for cardiovascular disease management in primary health care: systems for monitoring. Geneva: World Health Organization; 2018 (WHO/NMH/NVI/18.5). Licence: CC BY-NC-SA 3.0 IGO. WHO is not responsible for the content or accuracy of this translation. In the event of any inconsistency between the English and the Khmer translation, the original English version shall be the binding and authentic version.*

## A10. Health facility report

The *health facility report* is a suggested template for use by clinic staff in reporting quarterly and annual treatment enrolment and outcomes to district/provincial-level managers; such information will be collated from the *facility register for monitoring outcomes (Tool A9)*. The template can also be used to report quarterly quantities of drugs available and requested quantities for the next quarter. District and/or provincial-level managers can use this information to monitor progress, revisit current management practices as and when needed and submit reports for collating information at the national level.

| HEALTH FACILITY REPORT | | |
| --- | --- | --- |
| *Sections A and B will be filled out by health facilities where HT Facility Registers are placed.*  *Sections C and D will be filled out by all health facilities.* | | |
| Name of health facility: | **Name of district:** | |
| Name of province: | **Date of reporting:** | |
| Quarter and year for which you are making the report:  Quarter:  Year:  *(This is the ‘Reporting Quarter’. Usually this is the most recent quarter that has just finished.)* | | |
|  | | |
| Section A: Quarterly treatment enrolment and outcomes | | **Number of patients** |
| A1: Number of patients registered two quarters earlier: | |  |
| A2: Out of (A1), number of patients whose BP was documented to be <140/90 mmHg in the Reporting Quarter | |  |
|  | | |
| Section B: Annual treatment enrolment and outcomes  *(To be filled in only once a year, with Quarter 1 report):* | | **Number of patients** |
| B1: Number of patients whose BP is documented as <140/90 mmHg during Quarter 1. *(If the patient made more than one visit in the quarter, use most recent reading.)* | |  |
| B2: Estimated number of people in the catchment population *(only for district level):* | |  |
|  | | |
| Section C: Drug consumption and availability | | |
| Quarterly consumption of drugs *(give number of tablets)* | **Quantity of drugs available at the health facility *(give number of tablets)*** | **Quantity of drugs requested for the next quarter *(give number of tablets)*** |
| calcium channel blocker |  |  |
| angiotensin receptor blocker |  |  |
| angiotensin converting enzyme inhibitor |  |  |
| thiazide/thiazide like diuretic |  |  |
| Statin |  |  |
| Aspirin |  |  |
| beta blocker |  |  |
| Section D: Quarterly supervision | | |
| Was there a supervision visit to this health facility by district staff during the reporting quarter? | | Y / N |

*Reproduced and translated into Khmer from the HEARTS Technical package for cardiovascular disease management in primary health care: systems for monitoring. Geneva: World Health Organization; 2018 (WHO/NMH/NVI/18.5). Licence: CC BY-NC-SA 3.0 IGO. WHO is not responsible for the content or accuracy of this translation. In the event of any inconsistency between the English and the Khmer translation, the original English version shall be the binding and authentic version.*

## A11. Patient report card

The *patient report card* is filled in by a district/provincial-level manager to assess a health centre (HC)’s adherence to the treatment protocols through interviews with a random sample of patients visiting the HC on a given day. The specific questions asked can be modified based on the protocols applied in respective HCs. Information from this card will feed into a more comprehensive *treatment supervision/audit form (Tool A12)* completed by the district/provincial-level assessor.

| PATIENT REPORT CARD | | | | | | | |
| --- | --- | --- | --- | --- | --- | --- | --- |
| Name of health facility: | | **Name of district:** | | | | | |
| Name of province: | | **Date of reporting:** | | | | | |
| Name of supervisor: | | | | | | | |
| *Interview 5 patients and write yes or no for each question. To calculate the total, simply tally the number of yeses in each row. Copy the total into the clinical audit tool.* | | | | | | | |
| N | **Items** | | **Pt 1** | **Pt 2** | **Pt 3** | **Pt 4** | **Pt 5** |
| 1 | Did the patient receive all prescribed medicines at this visit? | |  |  |  |  |  |
| 2 | Did the patient ever have to pay for medicines in the past 6 months? | |  |  |  |  |  |
| 3 | Does the patient have a correct understanding of how to take medicines? | |  |  |  |  |  |
| 4 | Did the patient know whether their BP was under control at the last visit? | |  |  |  |  |  |
| 5 | Does the patient know the target BP? | |  |  |  |  |  |
| Signature of supervisor: | | | | | | | |

*Reproduced and translated into Khmer from the HEARTS Technical package for cardiovascular disease management in primary health care: systems for monitoring. Geneva: World Health Organization; 2018 (WHO/NMH/NVI/18.5). Licence: CC BY-NC-SA 3.0 IGO. WHO is not responsible for the content or accuracy of this translation. In the event of any inconsistency between the English and the Khmer translation, the original English version shall be the binding and authentic version.*

## A12. Treatment supervision/audit form

The *treatment supervision/audit form* is filled in by a district/provincial-level manager to assess a health centre (HC)’s adherence to treatment and administrative protocols. The specific questions asked can be modified based on the protocols applied in respective HCs.

| TREATMENT SUPERVISION / AUDIT FORM | | | |
| --- | --- | --- | --- |
| Name of health facility: | | **Name of district:** | |
| Name of province: | | **Date of reporting:** | |
| Name of supervisor: | | **Name of medical officer:** | |
| N | **Indicator** | **Tick any:** | **Remarks:** |
| 1 | **Human resources** |  |  |
| 1.1 | Are there dedicated health staff to provide NCD services? | Y N NA |  |
| 1.2 | Have dedicated health staff been appropriately trained? | Y N NA |  |
| 1.3 | Is there in-service training/NCD knowledge sharing to the HC team? | Y N NA |  |
| 1.4 | Has a physician from the RH provided technical backup at the HC in the last 3 months? | Y N NA |  |
| 2.1 | **Services: BP management** |  |  |
| 2.1.1 | Is opportunistic screening done for all adults? | Y N NA |  |
| 2.1.2 | Is the BP measurement protocol displayed on the wall/desk? | Y N NA |  |
| 2.1.3 | Are all patients with BP ≥ 140/90 referred to the medical officer for treatment? | Y N NA |  |
| 2.1.4 | For how many BP patients was BP measured correctly? *(Observe 5, >2 of each staff who measure BP.)* |  |  |
| 2.1.5 | Is the treatment algorithm displayed on the wall/desk? | Y N NA |  |
|  | ***Randomly audit 10 patient treatment cards (see Patient card audit form). Write for what proportion of patients:*** | Proportion: |  |
| 2.1.6 | BP was recorded at every visit for the last three visits | % |  |
| 2.1.7 | Initial antihypertensive medication was given as per protocol | % |  |
| 2.1.8 | Medication was intensified or added as per protocol if BP ≥ 140/90 | % |  |
| 2.1.8 | Aspirin was given if patient had prior CVD (write NA if not applicable) | % |  |
| 2.1.9 | Statin was given if patient >40 years with diabetes or if patient had prior CVD | % |  |
| 2.1.10 | Referral to a specialist was made if BP ≥ 140/90 after treating with three drugs | % |  |
| 2.1.11 | BP was <140/90 at last visit | % |  |
| 2.2 | **Services: DM management** |  |  |
| 2.2.1 | Is opportunistic screening done for those with suspected elevated blood sugar or diabetes**?** | Y N NA |  |
| 2.2.2 | Is the DM measurement protocol displayed on the wall/desk? | Y N NA |  |
| 2.2.3 | Are all patients with suspected elevated blood sugar or diabetes referred to the RH for treatment? | Y N NA |  |
| 2.2.4 | For how many DM patients was blood sugar measured correctly? *(Observe 5, >2 of each staff who measure BP.)* | Number: |  |
| 2.2.5 | Is the treatment algorithm displayed on the wall/desk? | Y N NA |  |
|  | ***Randomly audit 10 patient treatment cards (see Patient card audit form). Write for what proportion of patients:*** | Proportion: |  |
| 2.2.6 | Blood sugar levels was tested at every visit for the last three visits | % |  |
| 2.2.7 | Referral to the RH was made if patient had suspected elevated blood sugar or diabetes | % |  |
| 2.3 | **Services: Counselling** |  |  |
| 2.3.1 | Are patient counselling tools/materials available? | Y N NA |  |
| 2.3.2 | Have patients received health education and counselling on NCD risk factors? | Y N NA |  |
| 2.3.3 | Have all eligible patients been screened for CVD risk? | Y N NA |  |
| 2.4 | **Services: Patient interviews** *(see Patient interview report card)* |  |  |
| 2.4.1 | Number of interviews where BP measured at every visit | Number: |  |
| 2.4.2 | Number of interviews where the patient receive all prescribed medicines at this visit? | Number: |  |
| 2.4.3 | Number of interviews where the patient had to pay for medicines in the past? | Number: |  |
| 2.4.4 | Number of interviews where the patient had a correct understanding of how to take medicines | Number: |  |
| 2.4.5 | Number of interviews where the patient knew his/her BP reading at this visit | Number: |  |
| 2.4.6 | Number of interviews where the patient knew the target BP? | Number: |  |
| 3 | **Record keeping/MIS** |  |  |
| 3.1 | Is there a functioning recording and reporting system in place? | Y N NA |  |
| 3.2 | Are NCD patients recorded in a HC registration book? | Y N NA |  |
| 3.3 | Has the patient registration form been used appropriately? | Y N NA |  |
| 3.4 | Has the patient follow-up form been used appropriately? | Y N NA |  |
| 3.5 | Are patient’s self-management books available and in use? | Y N NA |  |
| 3.6 | Are there sufficient patient cards for next three months? | Y N NA |  |
| 3.7 | Is there a place to arrange/store patient cards? | Y N NA |  |
| 3.8 | Are the cards organized by serial number or other system so easily retrievable? | Y N NA |  |
| 3.9 | Was last quarter’s report sent on time? | Y N NA |  |
| 3.10 | Does the clinic in charge know the percentage of patients with BP <140/90 at the facility? | Y N NA |  |
| 3.11 | Is the last quarter’s 6-month BP control rate reported accurately? *(Check register from last quarter.)* | Y N NA |  |
| 4 | **Referral system** |  |  |
| 4.1 | Is the referral form used in the process of referring patient from HC to RH? | Y N NA |  |
| 4.2 | Have any patients been referred upward to RH, according to guidelines, in the last three months? | Y N NA |  |
| 4.3 | Have any patients been referred from RH to the HC, according to guidelines, in the last three months? | Y N NA |  |
| 4.4 | Is there a mechanism in place to follow-up the referred patient from HC to RH and RH to HC? | Y N NA |  |
| 5 | **Medicines and materials/equipment** |  |  |
| 5.1 | Is the printed National SOP for Hypertension and Diabetes Management in Primary Care available | Y N NA |  |
| 5.2 | Are glucometer and blood glucose strips available and functioning? | Y N NA |  |
| 5.3 | Are the blood pressure measurement devices available and functioning? | Y N NA |  |
| 5.4 | Is the CVD risk prediction chart and IEC materials for diabetes and hypertension, and other NCDs available? | Y N NA |  |
| 5.5 | Are NCD medicines for use at the HC level available? | Y N NA |  |
| 5.6 | Has the HC experienced out-of-stock of any antidiabetic and anti-hypertensive medicines in the last 3 months? | Y N NA |  |
| 5.7 | If there was a stock-out this quarter, which drugs were not available? | Y N NA |  |
| 5.8 | Is there enough buffer stock of core drugs for the next quarter? | Y N NA |  |
| 6 | **Community link** | Y N NA |  |
| 6.1 | Has the HC conducted awareness raising on NCDs and screened for hypertension and diabetes in the community in the last 3 months? | Y N NA |  |
| 6.2 | Have VHSG and/or PEs reported on follow-up of patients with diabetes/HBP in the last 3 months? | Y N NA |  |
| 6.3 | Are there minutes for PEN team meetings in the last 3 months? | Y N NA |  |
| 6.4 | Have any patients been referred by the VHSG and/or PE for CVD risk screening in the last 3 months? | Y N NA |  |
| 6.5 | Are there any contracted peer educators? | Y N NA |  |
| 7 | **Finance and administration** | Y N NA |  |
| 7.1 | Is the PEN team established officially? | Y N NA |  |
| 7.2 | Are user fees set for hypertension and diabetes? | Y N NA |  |
| 7.3 | Are requested procedures for medicines and materials/equipment followed? | Y N NA |  |
| 7.4 | Does the HC have a dashboard containing data on the number of patients with hypertension or diabetes? | Y N NA |  |
| Signature of supervisor: | |  |  |

*Adapted from: Ministry of Health (2019). National Standard Operating Procedure for Diabetes and Hypertension Management in Primary Care. Department of Preventive Medicine. Phnom Penh, Ministry of Health and HEARTS Technical package for cardiovascular disease management in primary health care: systems for monitoring. Geneva: World Health Organization; 2018 (WHO/NMH/NVI/18.5). Licence: CC BY-NC-SA 3.0 IGO.*

## A13. Summary of supervision visits

Based on information recorded in the *patient report card (Tool A11)*  and *treatment supervision/audit form (Tool A12)*, the *summary of supervision visits form* *(Tool A13)* is a template for use by a district/provincial-level manager to summarise the main problems identified in service delivery and administrative management of a HC and provide recommendations for the same.

| SUMMARY OF SUPERVISION VISITS | | |
| --- | --- | --- |
| Name of health facility: | | **Name of district:** |
| Name of province: | | **Date of reporting:** |
| Name of supervisor: | | |
| N | **Problem identified** | **Recommendations** |
| 1 | **Screening and BP measurement** |  |
| 2 | **Treatment** |  |
| 3 | **Counselling and follow-up** |  |
| 4 | **Service delivery including costs to patient** |  |
| 5 | **Drug inventory** |  |
| 6 | **Recording and reporting** |  |
| 7 | **Any other** |  |
| Signature of supervisor: | | |

*Reproduced and translated into Khmer from the HEARTS Technical package for cardiovascular disease management in primary health care: systems for monitoring. Geneva: World Health Organization; 2018 (WHO/NMH/NVI/18.5). Licence: CC BY-NC-SA 3.0 IGO. WHO is not responsible for the content or accuracy of this translation. In the event of any inconsistency between the English and the Khmer translation, the original English version shall be the binding and authentic version.*

## A14. Pre-visit planning: Workflow mapping worksheet

A *pre-visit planning workflow mapping sheet* is filled in by clinic staff and district/provincial-level managers to identify and document best practices for planning pre-scheduled visits with patients, assess current practices relative to best practices and document how the practice can be improved (where applicable) within given resource and other constraints. Such an exercise can include stakeholders such as healthcare workers at the community level, patients and patient advocacy groups, and policymakers at the district or provincial level, for example.

CARE TEAM:

Review reporting data to identify opportunities for quality improvement.

**Additional notes:**

Confirm upcoming appointments.

Prepare educational materials for upcoming patient appointments.

Contact high-risk patients and schedule appointments.

Send patients reminders for follow-up visits.

POTENTIAL PRACTICE

CARE TEAM:

CURRENT PRACTICE

Run list of patients with chronic conditions.

Identify and prioritise high-risk patients.

BEST

PRACTICE

*Reproduced and translated into Khmer by the KHANA Centre for Population Health Research from the HEARTS Technical package for cardiovascular disease management in primary health care: systems for monitoring. Geneva: World Health Organization; 2018 (WHO/NMH/NVI/18.5). Licence: CC BY-NC-SA 3.0 IGO. WHO is not responsible for the content or accuracy of this translation. In the event of any inconsistency between the English and the Khmer translation, the original English version shall be the binding and authentic version.*

## A15. Pre-visit planning: Assessment of current practice

A *pre-visit planning assessment of current practice sheet* is filled in by clinic staff and district/provincial-level managers to assess and document in greater detail current practices for planning pre-scheduled visits with patients, identify barriers or bottlenecks in improving current processes and identify what the “ideal” or an improved process could be if said bottlenecks were addressed. Such an exercise can include stakeholders such as healthcare workers at the community level, patients, patient advocacy groups, and policymakers at the district or provincial level, for example.

| **Process** | **Current process** | **Barriers / duplication** | **Ideal process** |
| --- | --- | --- | --- |
| 1. Does the practice conduct any pre-visit planning? Describe the process. |  |  |  |
| **Registry and reporting** | **Current process** | **Barriers / duplication** | **Ideal process** |
| 1. How does the practice monitor and track patients with chronic conditions? |  |  |  |
| 1. Does the practice monitor at-risk populations? How is the physician alerted? |  |  |  |
| 1. What are the current reporting requirements for the practice (e.g., patient-specific populations, time studies, clinical operating reports, incoming referrals, patient-care measures)?   How frequently are these reports run? |  |  |  |
| 1. Does anyone at the practice conduct chart reviews? How frequently? Describe the process. |  |  |  |

*Reproduced and translated into Khmer by the KHANA Centre for Population Health Research from the HEARTS Technical package for cardiovascular disease management in primary health care: systems for monitoring. Geneva: World Health Organization; 2018 (WHO/NMH/NVI/18.5). Licence: CC BY-NC-SA 3.0 IGO. WHO is not responsible for the content or accuracy of this translation. In the event of any inconsistency between the English and the Khmer translation, the original English version shall be the binding and authentic version.*

**Additional notes:**

## A16. Patient visit: Workflow mapping worksheet

*A patient visit workflow mapping sheet* is filled in by clinic staff and district/provincial-level managers to identify and document best practices during a patient visit, assess current practices relative to best practices and document how the practice can be improved (where applicable) within given resource and other constraints. Such an exercise can include stakeholders such as healthcare workers at the community level, patients, patient advocacy groups and policymakers at the district or provincial level, for example.

MEDICAL ASSISTANT:

CARE TEAM:

Meet to discuss patients for the day. Identify and prioritise high-risk patients.

RECEPTION COUNTER:

Check in patient. Update contact information. Enter data.

MEDICAL ASSISTANT:

Collect vitals, update medications list. Assess medical history, allergies and social history. Assess adherence to medications.

BEST

PRACTICE

HEALTH CARE PROVIDER:

Reconcile medications with patient. Order required lab tests. Conduct examination. Develop care plan and treatment goals with patient. Review progress. Assess and address barriers. Prescribe medications. Provide counselling if applicable. Refer for additional care and/or self-management support.

CARE TEAM:

Discuss patients and needed follow-up at the end of the day.

RECEPTION COUNTER:

Schedule next appointment. Provide Clinical Visit Summary. Check out patient.

MEDICAL ASSISTANT:

Collect specimen for labs. Confirm lab and referral orders. Provide patient resources.

CARE TEAM:

RECEPTION COUNTER:

CURRENT

PRACTICE

HEALTH CARE PROVIDER:

CARE TEAM:

MEDICAL ASSISTANT:

RECEPTION COUNTER:

CARE TEAM:

POTENTIAL PRACTICE

**Additional notes:**

MEDICAL ASSISTANT:

RECEPTION COUNTER:

HEALTH CARE PROVIDER:

CARE TEAM:

MEDICAL ASSISTANT:

RECEPTION COUNTER:

*Reproduced and translated into Khmer by the KHANA Centre for Population Health Research from the HEARTS Technical package for cardiovascular disease management in primary health care: systems for monitoring. Geneva: World Health Organization; 2018 (WHO/NMH/NVI/18.5). Licence: CC BY-NC-SA 3.0 IGO. WHO is not responsible for the content or accuracy of this translation. In the event of any inconsistency between the English and the Khmer translation, the original English version shall be the binding and authentic version.*

## A17. Patient visit: Assessment of current practice

A *patient visit assessment of current practice sheet* is filled in by clinic staff and district/provincial-level managers to assess and document in greater detail current practices during a patient visit, identify barriers or bottlenecks in improving current processes and identifying what the “ideal” or an improved process could be if said bottlenecks are addressed. Such an exercise can include stakeholders such as healthcare workers at the community level, patients, patient advocacy groups and policymakers at the district or provincial level, for example.

| **Rooming the patient** | **Current process** | **Barriers / duplication** | **Ideal process** |
| --- | --- | --- | --- |
| 1. What is the average patient wait time to see the physician? |  |  |  |
| 1. How does the front office notify the medical assistant that the patient is ready to be taken back? |  |  |  |
| **Vitals/intake** | **Current process** | **Barriers / duplication** | **Ideal process** |
| 1. Does the practice have a triage room/area? Who conducts triage, where is it conducted, and how? What is measured? How is it recorded? |  |  |  |
| 1. Who conducts initial screenings, i.e., chief complaints, subjective history, etc.? |  |  |  |
| 1. Who reviews current medications in the medical record? Is it completed for every visit? |  |  |  |
| 1. Does the practice perform tobacco screening and cessation counselling for tobacco users? |  |  |  |
| **Provider** | **Current process** | **Barriers / duplication** | **Ideal process** |
| 1. What is the communication and handoff between the medical assistant and the provider (e.g. reviewing of vitals, concerns)? |  |  |  |
| 1. Review how the provider manages patients with chronic conditions, i.e. referrals, medication reconciliation and adherence, treatment procedures, etc. |  |  |  |
| 1. How does the provider communicate to the medical assistant that the patient is ready for checkout? What action is taken, i.e. bill given, lab orders, vaccines, etc.? |  |  |  |
| 1. How long does it take the provider to write visit notes? Does the provider complete during or after the visit? When does the provider sign-off the chart? |  |  |  |
| 1. Are prescriptions provided to the patient or sent directly to the pharmacy? |  |  |  |
| 1. Who gives the patient their prescriptions and associated prescription education (i.e. pharmacist or physician during visit)? |  |  |  |
| 1. Who provides patient education? How (i.e. paper, electronic)? |  |  |  |
| **Referrals** | **Current process** | **Barriers / duplication** | **Ideal process** |
| 1. Do you have a list of providers you commonly refer patients to? |  |  |  |
| **Check out** | **Current process** | **Barriers / duplication** | **Ideal process** |
| 1. How does the patient get directed to check out? Describer the check-out process. |  |  |  |
| 1. Is a follow-up visit scheduled? Are instructions / education provided? |  |  |  |
| 1. Does the practice provide the patient with any forms during check-out? |  |  |  |

*Reproduced and translated into Khmer by the KHANA Centre for Population Health Research from the HEARTS Technical package for cardiovascular disease management in primary health care: systems for monitoring. Geneva: World Health Organization; 2018 (WHO/NMH/NVI/18.5). Licence: CC BY-NC-SA 3.0 IGO. WHO is not responsible for the content or accuracy of this translation. In the event of any inconsistency between the English and the Khmer translation, the original English version shall be the binding and authentic version.*

**Additional notes:**

## A18. Post-visit follow-up: Workflow mapping sheet

A *post-visit planning workflow mapping worksheet* is filled in by clinic staff and district/provincial-level managers to identify and document best practices for planning post-visit follow-ups with patients, assess current practices relative to best practices and document how the practice can be improved (where applicable) within given resource and other constraints. Such an exercise can include stakeholders such as healthcare workers at the community level, patients, patient advocacy groups and policymakers at the district or provincial level, for example.

**Additional notes:**

*Reproduced and translated into Khmer by the KHANA Centre for Population Health Research from the HEARTS Technical package for cardiovascular disease management in primary health care: systems for monitoring. Geneva: World Health Organization; 2018 (WHO/NMH/NVI/18.5). Licence: CC BY-NC-SA 3.0 IGO. WHO is not responsible for the content or accuracy of this translation. In the event of any inconsistency between the English and the Khmer translation, the original English version shall be the binding and authentic version.*

PRACTICE ADMIN:

Confirm appropriate billing documentation.

FRONT DESK:

Track referrals. Complete patient follow-up reminder. Respond to patient messages. Escalate urgent messages to provider.

HEALTH CARE PROVIDER:

Review laboratory results. Contact patients with abnormal results. Respond to patient messages.

BEST

PRACTICE

PRACTICE ADMIN:

FRONT DESK:

HEALTH CARE PROVIDER:

CURRENT PRACTICE

PRACTICE ADMIN:

FRONT DESK:

HEALTH CARE PROVIDER:

POTENTIAL PRACTICE

## A19. Post-visit follow-up: Assessment of current practice

A *pre-visit planning assessment of current practice sheet* is filled in by clinic staff and district/provincial-level managers to assess and document in greater detail current practices for planning post-visit follow-ups with patients, identify barriers or bottlenecks in improving current processes and identifying what the “ideal” or an improved process could be if said bottlenecks are addressed. Such an exercise can include stakeholders such as healthcare workers at the community level, patients, patient advocacy groups and policymakers at the district or provincial level, for example.

| **Referrals** | **Current process** | **Barriers / duplication** | **Ideal process** |
| --- | --- | --- | --- |
| 1. How has the loop closed to ensure continuity of care (e.g., nurse, medical assistant, practice administrator)? |  |  |  |
| 1. Does the practice common generate outgoing referrals? Receive incoming referrals? Which is more common? Describe overall processes. |  |  |  |
| **Clinical telephone encounters** | **Current process** | **Barriers / duplication** | **Ideal process** |
| 1. What are the most telephone encounters (e.g.: refills, test results, referrals)? Describe the process. Are there specific policies in place regarding turnaround time? |  |  |  |
| 1. How are incoming calls tracked (i.e., is a telephone encounter created for each call)? |  |  |  |
| 1. What is the process for returning calls? Outgoing calls (e.g., lab results)? |  |  |  |
| **Clinical telephone encounters** | **Current process** | **Barriers / duplication** | **Ideal process** |
| 1. Does the provider offer after-hours or weekend calls? How are these documented? Describe the process. |  |  |  |

*Reproduced and translated into Khmer by the KHANA Centre for Population Health Research from the HEARTS Technical package for cardiovascular disease management in primary health care: systems for monitoring. Geneva: World Health Organization; 2018 (WHO/NMH/NVI/18.5). Licence: CC BY-NC-SA 3.0 IGO. WHO is not responsible for the content or accuracy of this translation. In the event of any inconsistency between the English and the Khmer translation, the original English version shall be the binding and authentic version.*

**Additional notes:**

# References

Ministry of Health (2013). National Strategic Plan for the Prevention and Control of Noncommunicable Diseases: 2013-2020. Phnom Penh, Cambodia, Ministry of Health.

Ministry of Health (2018). National Multisectoral Action Plan for the Prevention and Control of Noncommunicable Diseases. Phnom Penh, Cambodia, Ministry of Health.

Ministry of Social Affairs, Veterans and Youth Rehabilitation. (2017). National Ageing Policy 2017-2030. Phnom Penh, Cambodia, Ministry of Social Affairs, Veterans and Youth Rehabilitation.

National Institute of Statistics. (2020). General Population Census of the Kingdom of Cambodia, 2019. *Ministry of Planning*.

National Institute of Statistics/Cambodia. (2015). Cambodia Demographic and Health Survey 2014. Phnom Penh, Cambodia, National Institute of Statistics/Cambodia, Directorate General for Health/Cambodia, and ICF International.

PL Annear, et al. (2015). The Kingdom of Cambodia Health System Review. Health Systems in Transition Bart Jacobs and Matthias Nachtnebel. Geneva, World Health Organization (on behalf of the Asia Pacific Observatory on Health Systems and Policies).

University of Health Sciences. (2010). Prevalence of Non-Communicable Disease Risk Factors in Cambodia: STEPS Survey Country Report, September 2010. Phnom Penh, Cambodia, University of Health Sciences.

University of Health Sciences. (2017). Prevalence of Non-Communicable Disease Risk Factors in Cambodia (STEPS Survey 2016). Phnom Penh, Cambodia, University of Health Sciences.

Uta Lehmann, D. S. (2007). Community health workers: What do we know about them? Evidence and Information for Policy. Geneva, Department of Human Resources for Health.

World Health Organization (2018). Global Health Estimates 2016: Deaths by Cause, Age, Sex, by Country and by Region, 2000-2016. Geneva, World Health Organization.

World Health Organization (2018). HEARTS Technical package for cardiovascular disease management in

primary health care: implementation guide. Geneva, WHO.

World Health Organization (2020). WHO package of essential noncommunicable (PEN)

disease interventions for primary health care. Geneva, World Health Organization.

1. Community health workers are defined as “members of the communities where they work, should be selected by the communities, should be answerable to the communities for their activities, should be supported by the health system but not necessarily a part of its Organisation, and have shorter training than professional workers” in 10. Uta Lehmann, D.S., *Community health workers: What do we know about them?*, in *Evidence and Information for Policy*. 2007, Department of Human Resources for Health: Geneva. [↑](#footnote-ref-2)
2. Each tool will be filed in either a clinic registration file, patient record or held by the patient themselves. The patient record would be subdivided into the following categories: (1) administration, (2) care encounters [including missed visits and referral consultations], and (3) laboratory. [↑](#footnote-ref-3)
3. ^1^ Example administrative tools are listed in the Appendix. [↑](#footnote-ref-4)
4. Lost-to-follow-up: defined as incontactable in the last 12 months [↑](#footnote-ref-5)
5. Lost-to-follow-up: defined as incontactable in the last 12 months [↑](#footnote-ref-6)
